# Immune Checkpoint Signatures in Minimal Change Disease and Membranous Nephropathy: Divergent Pathways of a Shared Imbalance

**DOI:** 10.3390/ijms262311371

**Published:** 2025-11-25

**Authors:** Sebastian Mertowski, Paulina Mertowska, Milena Czosnek, Iwona Smarz-Widelska, Wojciech Załuska, Ewelina Grywalska

**Affiliations:** 1 Department of Experimental Immunology, Medical University of Lublin, Chodźki 4a Street, 20-093 Lublin, Poland; sebastian.mertowski@umlub.edu.pl (S.M.); ewelina.grywalska@umlub.edu.pl (E.G.); 2 Student Research Group of Experimental Immunology, Medical University of Lublin, Chodźki 4a Street, 20-093 Lublin, Poland; 3 Department of Nephrology, Cardinal Stefan Wyszynski Provincial Hospital in Lublin, Al. Kraśnicka Street, 20-718 Lublin, Poland; 4 Department of Nephrology, Medical University of Lublin, 8 Jaczewskiego Street, 20-954 Lublin, Poland; wojciech.zaluska@umlub.edu.pl

**Keywords:** glomerulonephritis, minimal change, glomerulonephritis, membranous, programmed cell death 1 receptor, programmed cell death 1 ligand 1 protein, cytotoxic t-lymphocyte antigen 4, CD86 antigens, ox-2 membrane glycoprotein, peripheral blood mononuclear cells, flow cytometry, biomarkers

## Abstract

Primary glomerulopathies share common immune dysregulation but differ in their predominant pathways. We compared immune checkpoint profiles in minimal change disease (MCD) and membranous nephropathy (MN) with those in healthy volunteers (HV). In a cohort of 90 individuals (MCD, *n* = 30; MN, *n* = 30; HV, *n* = 30), we performed multiparameter flow cytometry of PBMCs to assess the expression of PD-1/PD-L1, CTLA-4/CD86, and CD200/CD200R on CD4+and CD8+ T cells, CD19+ B cells, and natural killer cells (NK cells). ELISA measured serum soluble checkpoint concentrations, and transcript levels in PBMCs were measured by qPCR. Nonparametric statistics and ROC analysis were used. In MCD, a skewed T cell pattern was observed, characterized by dominant expression of PD-1 and CTLA-4, whereas in MN, a humoral predominance was observed with higher PD-L1 expression and attenuated CD200/CD200R axis. Across diseases, expression profiles and correlations between markers differed between HV and between MCD and MN. Soluble checkpoints (sPD-1, sPD-L1, sCD200, sCD200R) showed potential discriminatory value for GN compared to HV and for differentiating MCD from MN in ROC analyses. These findings indicate that the mechanisms maintaining immune tolerance in primary GN are standard but pathway-specific, consistent with the dominant immunological component of each disease. A significant implication of this study is the need to conduct tissue-level studies to confirm clinical utility and provide insights into personalized immunomodulatory strategies targeting PD-1/PD-L1, CTLA-4/CD86, and CD200/CD200R.

## 1. Introduction

Minimal change disease (MCD) and membranous nephropathy (MN) are the two most common primary glomerulopathies (GN) leading to nephrotic syndrome, but they differ in epidemiology, morphology, and immunological mechanisms [1,2,3]. MCD predominates in children but is also a significant cause of nephrotic syndrome in adults; it is characterized by sparse appearance on light microscopy and diffuse effacement of podocyte foot processes on electron microscopy, without immunological deposits [1,4,5,6]. The pathogenesis of MCD is largely functional: lymphocytic mediators (including Th2/Tfh signaling involving IL-13/IL-4) and Treg/Teff imbalance are believed to lower the threshold for podocyte damage, leading to proteinuria without permanent structural changes. Transient activation of costimulatory axes on podocytes (e.g., B7-1/CD80) and increased sensitivity to infectious and allergic stimuli have also been described [7,8,9,10]. Clinically, MCD is characterized by the sudden onset of nephrotic syndrome, often with normal blood pressure and normal glomerular filtration rate. The course may be recurrent, but the disease generally responds well to glucocorticosteroids. In cases of steroid dependence/resistance, calcineurin inhibitors or rituximab may be effective. Diagnosis is usually based on the clinical picture and response to treatment; biopsy is more often considered in adults or in atypical cases (e.g., the presence of hematuria, decreased GFR, steroid resistance) [5,11,12,13,14,15].

MN is the most common cause of nephrotic syndrome in adults and a prototype of autoimmune podocyte disease. The core pathogenesis is the in situ formation of subepithelial deposits along the glomerular basement membrane following a reaction against podocyte antigens, most commonly PLA2R (phospholipase A2 receptor M-type) and THSD7A, and less frequently NELL1 or other antigens. IgG4 antibodies predominate, and podocyte damage is exacerbated by complement activation (often via the alternative/lectin pathway). The disease can be primary (autoantibodies against PLA2R/THSD7A) or secondary (associated with factors such as cancer, infection, drugs, or autoimmune diseases); this distinction has prognostic and therapeutic implications [3,15,16,17,18]. The histopathological appearance of MN includes thickening of the glomerular capillary walls, granular deposits of IgG and complement components along the basement membrane, and characteristic “spikes” on silver staining; electron microscopy shows subepithelial deposits and secondary alterations of the basement membrane [16,19]. Clinically, MN presents with massive proteinuria, hypoalbuminemia, and edema; blood pressure may be elevated, and the risk of venous thrombosis is significant with significant protein loss [11,20]. Circulating biomarkers (anti-PLA2R, anti-THSD7A) and antigen staining in biopsy specimens facilitate diagnosis and monitoring of activity (a decrease in titer often precedes remission of proteinuria) [21,22]. Treatment includes nephroprotection (RAASi, SGLT2, thrombosis prophylaxis in selected cases) and immunosuppression tailored to the risk of progression (rituximab, cyclophosphamide-steroid regimens, calcineurin inhibitors). The natural history is variable: some patients achieve spontaneous remission, but others experience persistent proteinuria and progressive loss of renal function [3,23,24,25,26,27,28].

The common denominator of both diseases is the dependence of the integrity of the glomerular filtration barrier on a precisely regulated immune response [1,29,30]. MCD is dominated by reversible, functional podocyte disorders with an immune-mediated basis, without fixed deposits, whereas in MN, persistent, antibody-dependent autoimmunity against podocyte antigens plays a key role, the severity and duration of which determine the clinical course. These biological differences justify distinct diagnostic and therapeutic strategies and provide a basis for comparative analyses of the immune regulation profiles in both entities [1,11,31,32,33,34,35,36].

In primary GN, immune checkpoints play a crucial role, calibrating the threshold of lymphocyte reactivity and stabilizing peripheral and tissue tolerance [37,38]. The PD-1/PD-L1 axis inhibits TCR signaling and limits Tfh cell function, which reduces the maintenance of germline responses and autoantibody production. Failure of this axis promotes a humoral immune response, which is crucial in membranous nephropathy [39,40,41,42,43]. The CTLA-4/CD86 axis acts in the early phase of activation in lymph nodes through competition with CD28 and transendocytosis of B7 family ligands, enhancing Treg control over CD4+ and CD8+ T cells and, indirectly, over B cell activation [44,45,46]. Weakening this regulation lowers the activation threshold and facilitates the development of a chronic autoimmune response. The CD200R/CD200 axis forms a broad inhibitory network between lymphocytes, myeloid cells, NK cells, and tissues, raising the inflammatory threshold and suppressing the cytokine environment that promotes podocyte damage [42,47,48,49,50].

Our study aimed to compare the prevalence (frequency/surface expression level) of PD-1/PD-L1, CTLA-4/CD86, and CD200R/CD200 molecules on CD4+ T cells, CD8+ T cells, CD19+ B cells, and NK cells, as well as to compare the serum concentrations of these molecules and their expression levels in PBMCs between patients with MCD and MN.

## 2. Results

### 2.1. Clinical Characteristics of Patients Recruited into the Study

Ninety individuals were recruited for this study: 60 were patients with diagnosed, previously untreated primary glomerulonephritis (30 with MCD and 30 with MN), and 30 healthy volunteers (HV) served as controls. The median age of the recruited patients was 49.5 years for MCD patients, 57.50 years for MN patients, and 44.50 years for HV patients. Among the recruited patients, we recruited 15 women and 15 men in the MCD group, and 12 women and 18 men in the MN group. In the HV group, the gender mix was 13 women and 17 men. The first stage of our study involved analyzing selected hematological, biochemical, and renal function parameters in the recruited patients, which are summarized and presented in Table 1.

Compared to healthy volunteers, both MCD and MN groups of patients present with a typical picture of nephrotic syndrome, with very high proteinuria, decreased albumin and total protein levels, and marked dyslipidemia, including elevated total cholesterol, increased LDL, and higher triglycerides. The severity of these abnormalities is similar in both groups (Table 1). Renal function is poorer in both groups than in healthy volunteers, as evidenced by higher urea levels and lower eGFR. Filtration abnormalities are more severe in MCD, where eGFR is lower and urea is higher than in MN. Creatinine levels are elevated in MCD compared to healthy volunteers, while in MN, they do not differ significantly from control values. Uric acid levels are elevated in both patient groups compared to healthy volunteers, with the highest values observed in MCD (Table 1). Blood counts showed no significant differences between the groups. However, MCD had lower neutrophil counts than both MCD and healthy controls, and higher monocyte counts than MCD. Additionally, MCD had higher eosinophil counts than both the MCD and control groups. Both MCD and MN had lower erythrocyte counts and lower hemoglobin concentrations compared to healthy controls, while platelet counts and HDL levels remained unchanged across the groups. In the immunoglobulin profile, MCD had reduced IgG levels relative to healthy controls and MN. At the same time, MN had IgG levels similar to controls, and IgM levels were reduced in both MCD and MN compared to healthy controls, while IgA levels were normal (Table 1).

Analysis of the baseline lymphocyte composition of peripheral blood did not reveal significant differences between MCD, MN, and HV (Table 2). The percentage of CD45+ cells was high and comparable in all groups, confirming the absence of generalized leukopenia. The percentages of CD3+ T cells and CD19+ B cells were within similar ranges, with a significant overlap in interquartile ranges. The percentage of NK cells, defined as CD3-CD16+CD56+, did not differ significantly. Within the T-cell pool, the proportions of CD4+ and CD8+ cells were similar between groups, and the CD4/CD8 ratio remained comparable.

### 2.2. Upregulation of PD-1/PD-L1, CTLA-4/CD86, and CD200/CD200R Axes in MCD and MN Compared with Healthy Volunteers

The following steps in our study involved evaluating selected inhibitory immune checkpoint axes and their ligands—PD-1/PD-L1, CTLA-4/CD86, and CD200R/CD200—on T, B lymphocytes, and NK cells in the peripheral blood of recruited patients. Detailed data are presented in Supplementary Materials Table S1 and Figure S1.

**Figure 1:**
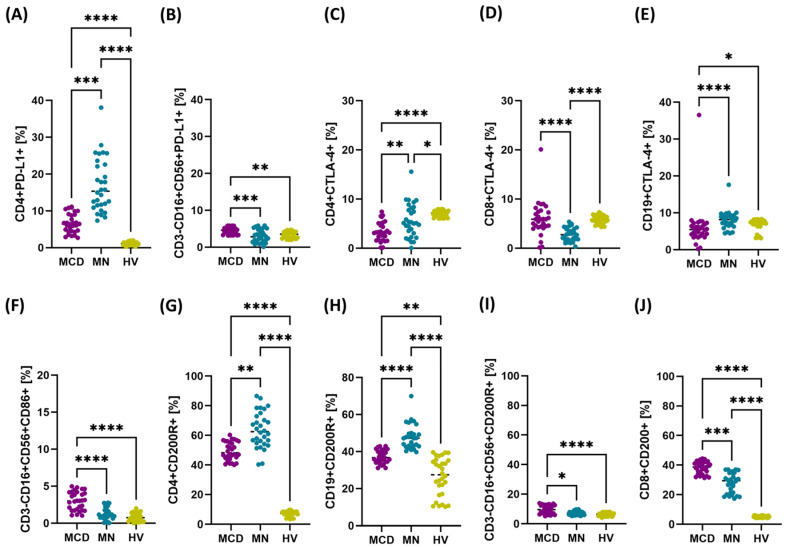
Percentage of PD-L1-, CTLA-4-, CD86-, CD200R- and CD200-positive cells in major lymphocyte and NK subpopulations in peripheral blood of newly diagnosed, untreated patients with minimal change disease (MCD) and membranous nephropathy (MN), and healthy volunteers (HV). Dot plots present individual values with medians indicated (horizontal line). Panels: (**A**) CD4+PD-L1+, (**B**) CD3-CD16+CD56+PD-L1+ (NK), (**C**) CD4+CTLA-4+, (**D**) CD8+CTLA-4+, (**E**) CD19+CTLA-4+, (**F**) CD3-CD16+CD56+CD86+ (NK), (**G**) CD4+CD200R+, (**H**) CD19+CD200R+, (**I**) CD3-CD16+CD56+CD200R+ (NK), (**J**) CD8+CD200+. Notations: * *p* < 0.05, ** *p* < 0.01, *** *p* < 0.001, **** *p* < 0.0001.

In the analysis of the first PD-1/PD-L1 axis in patients with GN, we found that the percentage of cells with positive PD-1 and PD-L1 expression was statistically significantly higher in both MCD and MN patients compared to HV. We did not observe significant differences in PD-1 expression between the studied disease subtypes in any of the analyzed immune cell subpopulations (Supplementary Materials Figure S1A–D). A significant increase in the percentage of PD-L1+ cells between MCD and MN was observed only for CD4+ T lymphocytes (higher in MN patients) and NK cells (higher in MCD patients) (Figure 1A,B).

The next axis analyzed was CTLA-4/CD86. Statistically significant differences were observed between patients with GN and HV in the percentage of CTLA-4+ cells among CD4+ T cells and NK cells, as well as CD86+ cells in all analyzed subpopulations (Supplementary Materials, Figure S1G–J). Between the MCD and MN groups, we demonstrated significant differences in CD4+CTLA-4+, CD8+CTLA-4+, and CD19+CTLA-4+ lymphocytes (Figure 1C–E). For CD86, the difference between disease subtypes reached statistical significance only for NK cells (Figure 1F).

The final axis analyzed concerned the expression of CD200R/CD200 in selected immune cell subpopulations. As with PD-1/PD-L1, we observed statistically significant differences between patients with GN and HV in all analyzed cell populations (Supplementary Materials Figure S1K–N). Between the MCD and MN groups, significant differences were found only for CD4+CD200R+, CD19+CD200R+ lymphocytes, CD3-CD16+CD56+CD200R+ NK cells, and CD8+CD200+ lymphocytes (Figure 1G–J).

**Figure 2:**
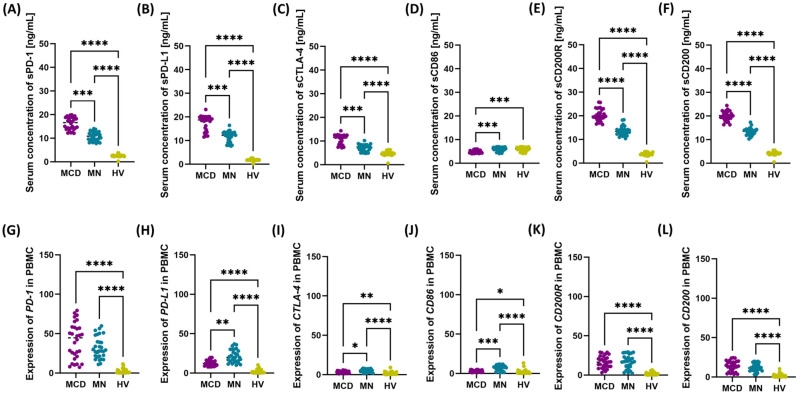
Serum concentrations and peripheral blood mononuclear cell (PBMC) expression of *PD-1*, *PD-L1*, *CTLA-4*, *CD86*, *CD200R* and *CD200* in newly diagnosed, untreated patients with minimal change disease (MCD) and membranous nephropathy (MN), and healthy volunteers (HV). Dot plots present individual values with medians indicated (horizontal line). Panels: (**A**) serum concentration of sPD-1, (**B**) serum concentration of sPD-L1, (**C**) serum concentration of sCTLA-4, (**D**) serum concentration of sCD86, (**E**) serum concentration of sCD200R, (**F**) serum concentration of sCD200, (**G**) expression of *PD-1* in PBMC, (**H**) expression of *PD-L1* in PBMC, (**I**) expression of *CTLA-4* in PBMC, (**J**) expression of *CD86* in PBMC, (**K**) expression of *CD200R* in PBMC, (**L**) expression of *CD200* in PBMC. Notations: * *p* < 0.05, ** *p* < 0.01, *** *p* < 0.001, **** *p* < 0.0001.

To complement the analyses, we also assessed serum concentrations of the studied immune checkpoints and their ligands in patients with GN and in the HV group. Detailed data are presented in the Supplementary Materials (Table S2) and Figure 2. We found significantly higher concentrations of soluble forms of the studied molecules in patients with GN compared to HV, with higher values in patients with MCD than in those with MN. Concentrations of sPD-1, sPD-L1, and sCTLA-4 were significantly elevated in both MCD and MN patients compared to HV, with values in MCD exceeding those in MN in each case (Figure 2A–C). We observed a different pattern for sCD86: concentrations in patients with MCD were lower than in patients with MN and HV, and statistical significance was confirmed by post hoc analyses, whereas values in MN and HV did not differ (Figure 2D). Both sCD200 and sCD200R levels were highest in patients with MCD, lower in patients with MN, and lowest in HV; these differences were significant for all pairwise comparisons (Figure 2E,F).

In addition to cellular phenotyping and serum measurements, transcript levels of genes encoding the studied checkpoints and their ligands were quantified in PBMC (Figure 2G–L). This analysis demonstrated significantly increased expression of the investigated genes in both types of GN compared with HV. In MN, transcript levels of *PD-L1*, *CTLA-4*, and *CD86* were higher than in MCD, while remaining elevated relative to HV. In MCD, the direction of change was analogous (increased values versus HV), although the magnitude of upregulation for these genes was lower than in MN. Transcript levels of *CD200* and *CD200R* were increased in both GN groups compared with HV and were similar between patients with MCD and MN (Figure 2K,L).

### 2.3. The Role of Gender in Modulating the Multilevel Profile of the PD-1/PD-L1, CTLA-4/CD86 and CD200/CD200R Axis—Cellular Expression, Soluble Forms and Transcripts in MCD and MN Compared to Healthy Volunteers

Recognizing sex as a biological variable reveals significant dimorphism in the immune response, which may modulate the clinical and molecular manifestations of primary glomerulopathies. Sex hormones (estrogens, progesterone, androgens), genetic and epigenetic factors (genes on the X chromosome, X-inactivation mosaicism, microRNA regulation), and differences in the maturation and aging of the immune system influence the activation and suppressive tone of T, B, and NK cells. In this context, the expression and release of molecules from the PD-1/PD-L1, CTLA-4/CD86, and CD200/CD200R axis may differ between women and men at the cellular, serum, and PBMC transcriptional levels, potentially impacting the severity of proteinuria, remission dynamics, risk of relapse, and response to immunomodulation. Because MCD is associated with a predominance of functional mechanisms (including the T/NK component) and MN with a sustained humoral immune response, analysis of sex differences may reveal distinct pathways of checkpoint regulation in these entities. Therefore, in the subsequent stages of our analysis, we compared the obtained results in the context of similarities and differences between the female and male patients recruited.

As described at the beginning, our analyses began by examining similarities and differences between the sexes in terms of basic laboratory parameters (Supplementary Materials, Table S4). Cross-sectionally, no systematic differences were found between women and men within the same disease entity (MCD or MN) for most hematological, biochemical, and lipid parameters. The basic picture—severe nephrotic syndrome with hypoalbuminemia, hypoproteinemia, dyslipidemia, hyperuricemia (especially in MCD), and impaired filtration—was common to both sexes. Gender differences, if any, were observed relative to the HV group and did not alter the general relationships previously presented.

After separating the groups into women and men, no significant differences were observed in the percentages of the main peripheral blood cell populations between MCD, MN, and healthy volunteers. The rate of CD45+ cells, the percentage of T cells (CD3+), B cells (CD19+), NK cells (CD3-CD16+CD56+), and the CD4+, CD8+, and CD4/CD8 ratios remained comparable both between genders within the same disease and between diseases within the same gender. The only exception was a lower CD3+ fraction in men with MCD compared to those in the control group, which reached statistical significance (*p* = 0.0177). Apart from this single signal, all other comparisons were nonsignificant, indicating that gender does not modify the macroscopic composition of the primary lymphocyte subpopulations at diagnosis (Supplementary Materials Table S5).

**Figure 3:**
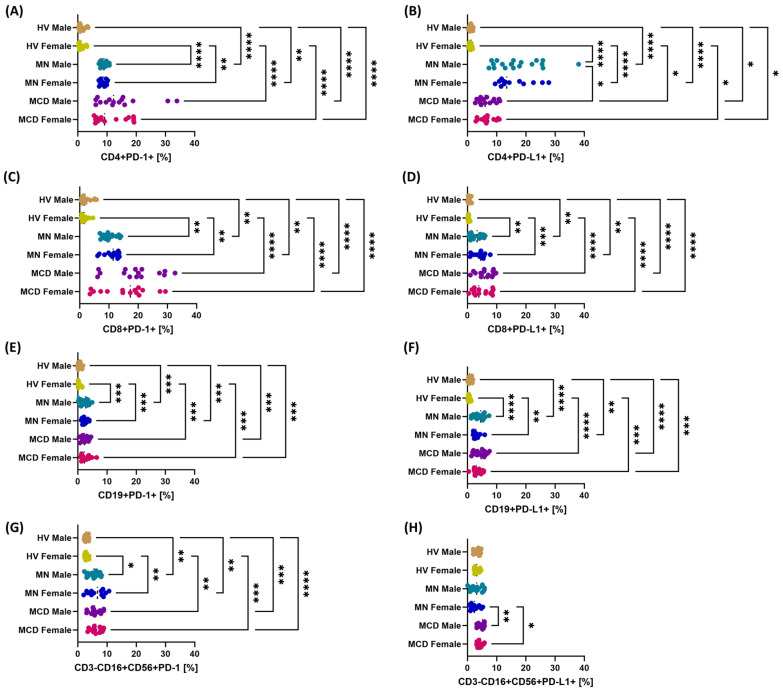
Percentages of PD-1- and PD-L1-positive cells in major lymphocyte and NK subpopulations after gender stratification in newly diagnosed, untreated patients with minimal change disease (MCD) and membranous nephropathy (MN), and in healthy volunteers (HV). The study groups comprised MCD females (*n* = 15), MCD males (*n* = 15), MN females (*n* = 12), MN males (*n* = 18), HV females (*n* = 13), and HV males (*n* = 17). Dot plots display individual values, with the median indicated by a dashed horizontal line. Panels: (**A**) CD4+PD-1+, (**B**) CD4+PD-L1+, (**C**) CD8+PD-1+, (**D**) CD8+PD-L1+, (**E**) CD19+PD-1+, (**F**) CD19+PD-L1+, (**G**) CD3-CD16+CD56+PD-1+ (NK), (**H**) CD3-CD16+CD56+PD-L1+ (NK). Symbols: * *p* < 0.05, ** *p* < 0.01, *** *p* < 0.001, **** *p* < 0.0001.

In both GN groups—in both women and men—the percentage of PD-1+ and PD-L1+ cells was significantly higher than in healthy volunteers in all analyzed subpopulations (Figure 3). Gender differences within the same disease entity were few and less significant than differences between patients and healthy individuals. Isolated gender-specific signals mainly concerned PD-L1 expression on CD4+ T lymphocytes (MCD men vs. MN men) and on NK cells (MCD women vs. MN women and MCD men vs. MN women) (Figure 3B,H). Regardless of gender, patients with MCD and MN exhibited precise and reproducible differences compared to healthy volunteers.

**Figure 4:**
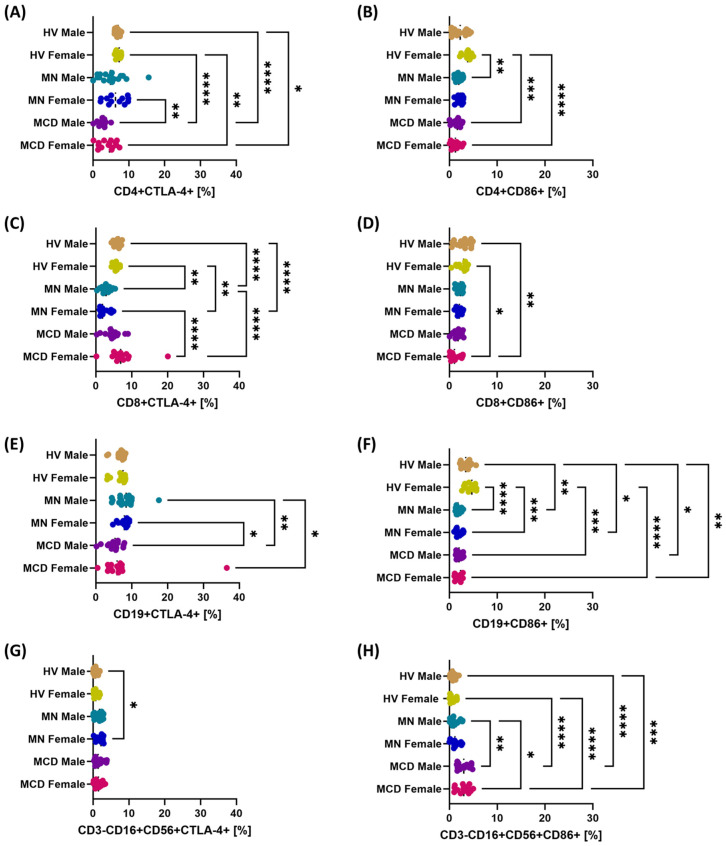
Percentages of cells positive for CTLA-4/CD86 axis molecules in major lymphocyte subpopulations and NK cells after gender stratification in newly diagnosed, untreated patients with minimal change disease (MCD) and membranous nephropathy (MN), and in healthy volunteers (HV). The study groups comprised MCD females (*n* = 15), MCD males (*n* = 15), MN females (*n* = 12), MN males (*n* = 18), HV females (*n* = 13) and HV males (*n* = 17). Dot plots display individual values, with the median indicated by a dashed horizontal line. Panels: (**A**) CD4+CTLA-4+, (**B**) CD4+CD86+, (**C**) CD8+CTLA-4+, (**D**) CD8+CD86+, (**E**) CD19+CTLA-4+, (**F**) CD19+CD86+, (**G**) CD3-CD16+CD56+CTLA-4+ (NK), (**H**) CD3-CD16+CD56+CD86+ (NK). Symbols: * *p* < 0.05, ** *p* < 0.01, *** *p* < 0.001, **** *p* < 0.0001.

On the CTLA-4 axis, the percentages of CD4+CTLA-4+ and CD19+CTLA-4+ T cells were higher in MCD patients than in MN patients and higher than in HV controls (Figure 4A,E). For CD8+ T cells, the percentages of CD8+CTLA-4+ T cells were lower in MN patients than in MCD patients and HV, and the values in MCD were similar to those observed in HV (Figure 4C). CTLA-4 expression on NK cells was low in all study groups, but the values in MCD and MN patients were higher than in HV; no significant differences were observed between MCD and MN (Figure 4G).

On the CD86 axis, the percentages of CD4+CD86+, CD8+CD86+, and CD19+CD86+ T cells were lower in GN patients than in healthy volunteers (Figure 4B,D,F). In the NK cell population, the percentage of CD3-CD16+CD56+CD86+ cells was highest in patients with MCD, lower in patients with MN, and lowest in HV (Figure 4H). Gender differences were observed in the percentage of CD4+CTLA-4+ cells (MCD men vs. MN women), CD8+CTLA-4+ cells (MCD women vs. MN women and MCD women vs. MN men), and CD19+CTLA-4+ cells (MCD men vs. MN women, MCD men vs. MN men, MCD women vs. MN men). Statistically significant gender differences were observed for CD86 in the NK cell population (MCD women vs. MN men and MCD men vs. MN men) (Figure 4H).

**Figure 5:**
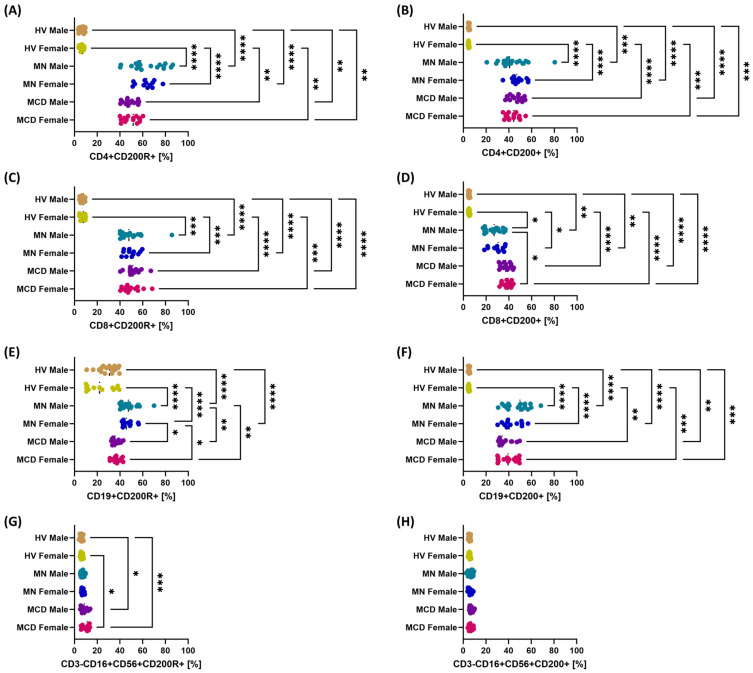
Percentages of cells positive for CD200R/CD200 axis molecules in major peripheral blood subpopulations after gender stratification in newly diagnosed, untreated patients with minimal change disease (MCD) and membranous nephropathy (MN), and in healthy volunteers (HV). The study groups comprised MCD females (*n* = 15), MCD males (*n* = 15), MN females (*n* = 12), MN males (*n* = 18), HV females (*n* = 13) and HV males (*n* = 17). Dot plots display individual values, with the median indicated by a dashed horizontal line. Panels: (**A**) CD4+CD200R+, (**B**) CD4+CD200+, (**C**) CD8+CD200R+, (**D**) CD8+CD200+, (**E**) CD19+CD200R+, (**F**) CD19+CD200+, (**G**) CD3-CD16+CD56+CD200R+ (NK), (**H**) CD3-CD16+CD56+CD200+ (NK). Symbols: * *p* < 0.05, ** *p* < 0.01, *** *p* < 0.001, **** *p* < 0.0001.

Regardless of gender, in patients with MCD and MN, the percentages of CD200R+ and CD200+ cells in all analyzed subpopulations were higher than in healthy volunteers (Figure 5). The most statistically significant gender differences in patients with GN concerned the percentage of CD19+CD200R+ B lymphocytes (MCD men vs. MN women; MCD women vs. MN women; MCD men vs. MN men; MCD women vs. MN men) (Figure 5E) and CD8+CD200+ lymphocytes (MCD women vs. MN men) (Figure 5D).

In women and men with GN, serum concentrations of sPD-1, sPD-L1, sCTLA-4, sCD200R, and sCD200 were higher compared to healthy controls, with the highest values observed in patients with MCD, intermediate values in patients with MN, and the lowest in HV. A different pattern was observed for sCD86: concentrations in patients with MCD (of both sexes) were lower than in patients with MN and in HV, while values in MN did not differ significantly from those in HV.

**Figure 6:**
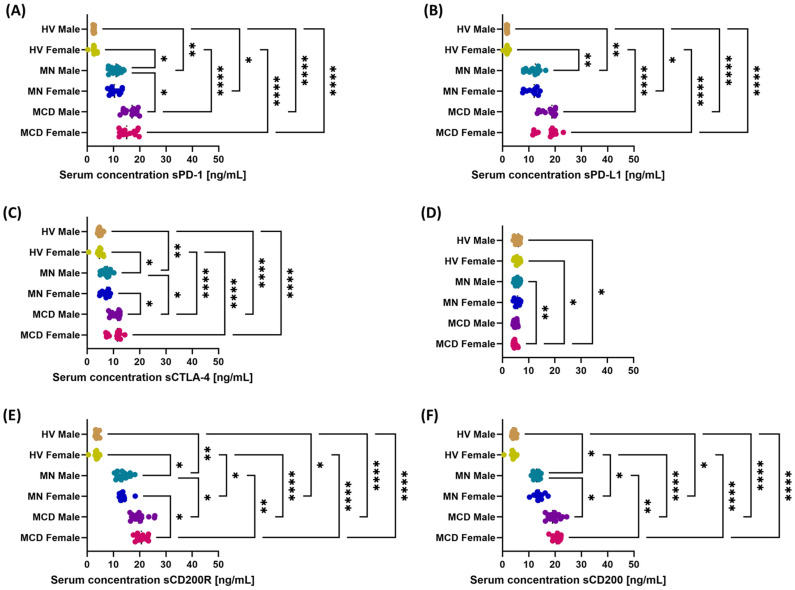
Serum concentrations of soluble immune checkpoints by gender in patients with minimal change disease (MCD) and membranous nephropathy (MN), and in healthy volunteers (HV). The study groups comprised MCD females (*n* = 15), MCD males (*n* = 15), MN females (*n* = 12), MN males (*n* = 18), HV females (*n* = 13), and HV males (*n* = 17). Dot plots display individual values, with the median indicated by a dashed horizontal line. Panels: (**A**) sPD-1, (**B**) sPD-L1, (**C**) sCTLA-4, (**D**) sCD86, (**E**) sCD200R, (**F**) sCD200. Symbols: * *p* < 0.05, ** *p* < 0.01, **** *p* < 0.0001.

Gender differences varied depending on the analyzed control point. Statistically significant differences were found for sPD-1 between men with MCD and men with MN (Figure 6A); for sCTLA-4 between men with MCD and women with MN, and between men with MCD and men with MN (Figure 6C); for sCD86 between females with MCD and males with MN (Figure 6D); for sCD200R between MCD and MN in females and between MCD and MN in males (Figure 6E); and for sCD200 between MCD and MN in males (Figure 6F).

**Figure 7:**
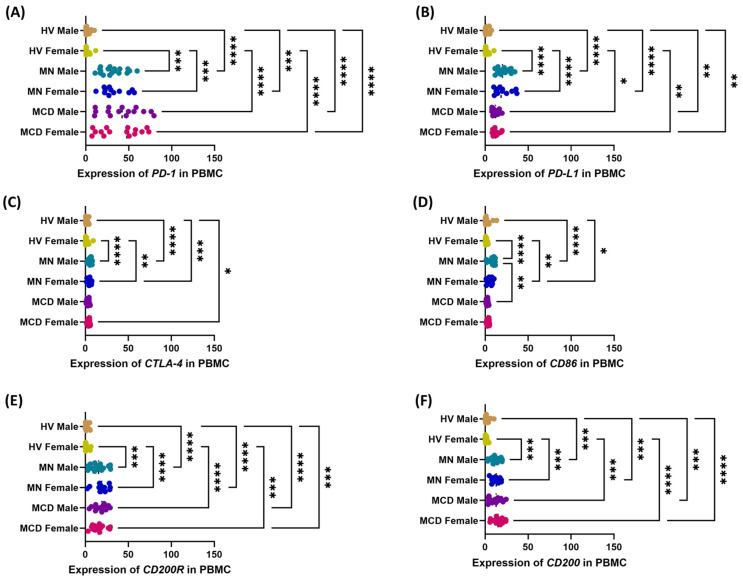
Expression of checkpoint transcripts in PBMC by gender in patients with minimal change disease (MCD) and membranous nephropathy (MN), and in healthy volunteers (HV). The study groups comprised MCD females (*n* = 15), MCD males (*n* = 15), MN females (*n* = 12), MN males (*n* = 18), HV females (*n* = 13) and HV males (*n* = 17). Dot plots display individual values, with the median indicated by a dashed horizontal line. Panels: (**A**) PD-1, (**B**) PD-L1, (**C**) CTLA-4, (**D**) CD86, (**E**) CD200R, (**F**) CD200. Symbols:, * *p* < 0.05, ** *p* < 0.01, *** *p* < 0.001, **** *p* < 0.0001.

A similar pattern of results was observed in analyses of the expression levels of the studied genes in PBMC (Figure 7). Compared with healthy controls, women and men with MCD and MN had significantly higher transcript levels of all analyzed markers. In MCD, regardless of gender, transcript levels of PD-L1, CTLA-4, and CD86 were higher than in MCD, whereas PD-1 levels were increased to a similar extent in both diseases, and CD200 and CD200R were elevated to comparable levels. Gender differences were also noted, particularly in CD86 expression between men with MCD and men with MN (Figure 7D).

### 2.4. Correlation Analysis and ROC Curves

To synthetically assess the biological consistency of the studied checkpoint axes and their diagnostic utility, we performed correlation analysis between cellular (PD-1/PD-L1, CTLA-4/CD86, CD200/CD200R percentages) and serum (soluble forms) parameters, as well as transcriptional parameters of PBMCs in three groups: MCD, MN, and HV. We used Spearman’s rank correlation and then assessed the ability to distinguish between groups using ROC curves. Due to the lack of significant gender-stratified correlations in the obtained results, we used pooled data, excluding this factor, for our analyses. Detailed results of these analyses are available in the Supplementary Materials Tables S6–S8.

**Figure 8:**
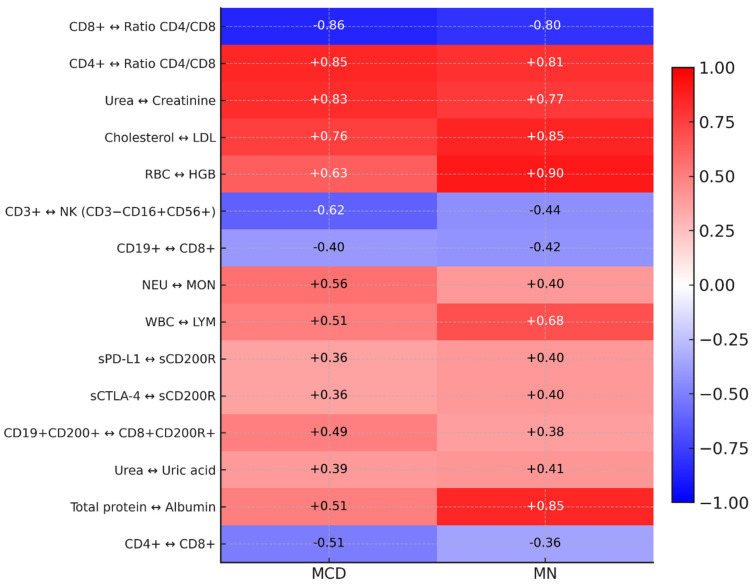
Heat map of common correlations (Spearman ranks) for pairs of variables assessed in MCD and MN. Scale color: red = positive correlation, blue = negative; values in cells are r (range −1 to 1).

In the case of correlation analyses of the tested immunological, biochemical, hematological, and clinical parameters, 150 statistically significant correlations were observed in patients with MCD, of which 55 were negative (1 very high; 1 high; 36 moderate, and 17 low) and 95 positive correlations (3 very high; 8 high; 56 moderate, and 28 low) (Supplementary Materials Table S6). In patients with MN, we observed 183 significant correlations, of which 72 were negative (3 high, 51 moderate, and 18 low) and 111 positive (4 very high, 11 high, 56 moderate, and 40 low) (Supplementary Materials Table S7). Detailed analysis revealed that some of the observed correlations are common to both patient groups, including the CD4+/CD8+ ratio pair (positive) and the CD8+/CD4+/CD8+ ratio pair (negative), a strong correlation between urea and creatinine, and positive associations in the lipid profile and the CD200/CD200R axis. In MN, some of the associations are clearly stronger than in MCD (e.g., RBC–HGB, cholesterol-LDL, WBC–LYM, total protein–albumin). At the same time, CD3+&NK antagonism maintains the same negative direction but is weaker than in MCD (Figure 8).

**Figure 9:**
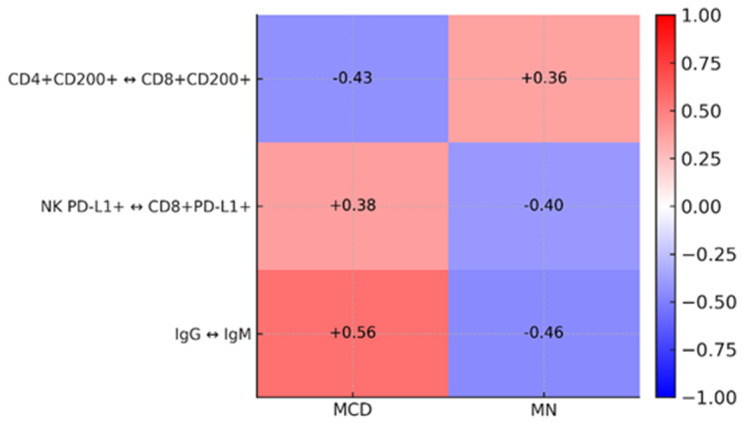
Heat map of the opposite-sign correlations between MCD and MN (Spearman rank). Scale color: red = positive correlation, blue = negative; values in cells are r (range −1 to 1).

Three pairs show an inverted direction of the relationship between MCD and MN: CD4+CD200+ andD8+CD200+ (MCD R = −0.43 vs. MN R = +0.36), NK PD-L1+andCD8+PD-L1+ (MCD R = +0.38 vs. MN T = −0.40), and IgG and IgM (MCD R = +0.56 vs. MN R = −0.46) (Figure 9). This indicates qualitatively different couplings between the CD200/CD200R axis, PD-L1 regulation on effector cells, and humoral immune response in MCD and MN.

**Figure 10:**
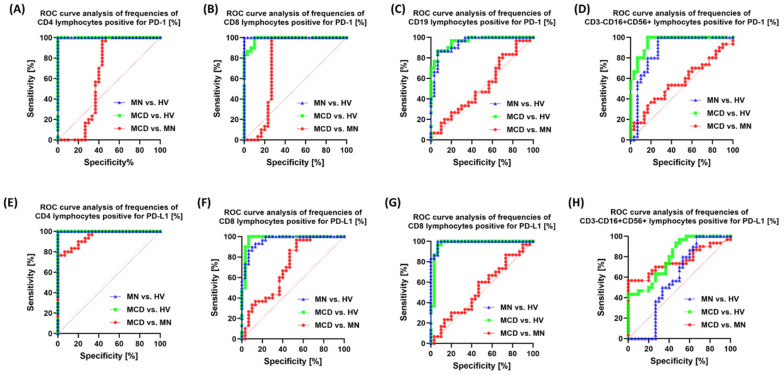
ROC curve analysis for the percentage of PD-1/PD-L1-positive cells in peripheral blood. Panels: (**A**) CD4+PD-1+, (**B**) CD8+PD-1+, (**C**) CD19+PD-1+, (**D**) CD3-CD16+CD56+PD-1+ (NK), (**E**) CD4+PD-L1+, (**F**) CD8+PD-L1+, (**G**) CD8+PD-L1+, (**H**) CD3-CD16+CD56+PD-L1+ (NK). Green—MCD vs. HV; blue—MN vs. HV; red—MCD vs. MN.

**Figure 11:**
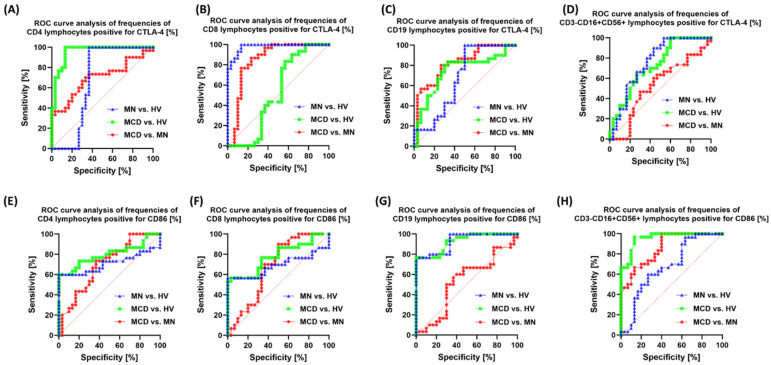
ROC curves for the percentages of CTLA-4 and CD86-positive cells in peripheral blood. Panels: (**A**) CD4+CTLA-4+, (**B**) CD8+CTLA-4+, (**C**) CD19+CTLA-4+, (**D**) CD3-CD16+CD56+CTLA-4+ (NK), (**E**) CD4+CD86+, (**F**) CD8+CD86+, (**G**) CD19+CD86+, (**H**) CD3-CD16+CD56+CD86+ (NK). Curve colors: green—MCD vs. HV, blue—MN vs. HV, red—MCD vs. MN.

**Figure 12:**
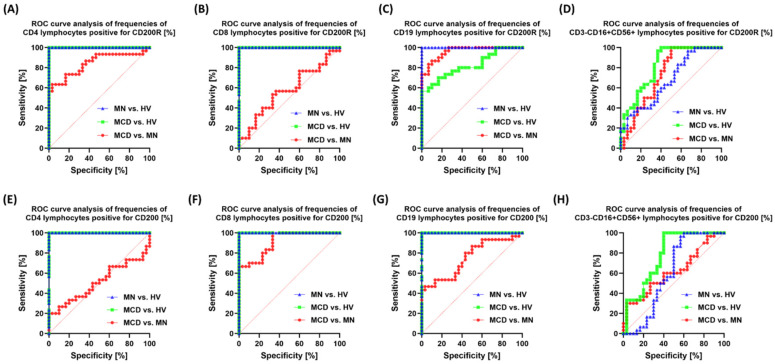
ROC curves for the percentages of CD200R and CD200-positive cells in peripheral blood. Panels: (**A**) CD4+CD200R+, (**B**) CD8+CD200R+, (**C**) CD19+CD200R+, (**D**) CD3-CD16+CD56+CD200R+ (NK), (**E**) CD4+CD200+, (**F**) CD8+CD200+, (**G**) CD19+CD200+, (**H**) CD3-CD16+CD56+CD200+ (NK). Curve colors: green—MCD vs. HV, blue—MN vs. HV, red—MCD vs. MN.

ROC analysis confirmed that the vast majority of markers distinguish patients (MCD/MN) from healthy individuals very well (AUC usually 0.95–1.00). At the same time, the MCD vs. MN distinction is selective and specific to individual markers. The strongest signals for MCD and MN differentiation were from: CD19+CD200R+ (AUC 0.96; *p* < 0.0001) (Figure 10), CD8+CD200+ (0.91; *p* < 0.0001) (Figure 10), CD4+PD-L1+ (0.95; *p* < 0.0001) (Figure 10), CD8+CTLA-4+ (0.85; *p* < 0.0001) (Figure 11), CD19+CTLA-4+ (0.82; *p* < 0.0001) (Figure 11) and NK CD86+ (0.86; *p* < 0.0001) (Figure 11). Also useful were CD8+PD-1+ (0.76; *p* = 0.0007) (Figure 10), NK PD-L1+ (0.76; *p* = 0.0005) (Figure 10), NK CD200R+ (0.73; *p* = 0.0021) (Figure 12) and CD19+CD200+ (0.75; *p* = 0.0008) (Figure 12). Markers with low resolving power MCD vs. MN included CD4+PD-1+ (0.63; *p* = 0.086), CD19+PD-1+ (0.52; *p* = 0.76), NK CTLA-4+ (0.53; *p* = 0.71), CD4+CD200+ (0.51; *p* = 0.88), NK CD200+ (0.62; *p* = 0.12) and CD8+CD200R+ (0.57; *p* = 0.33).

**Figure 13:**
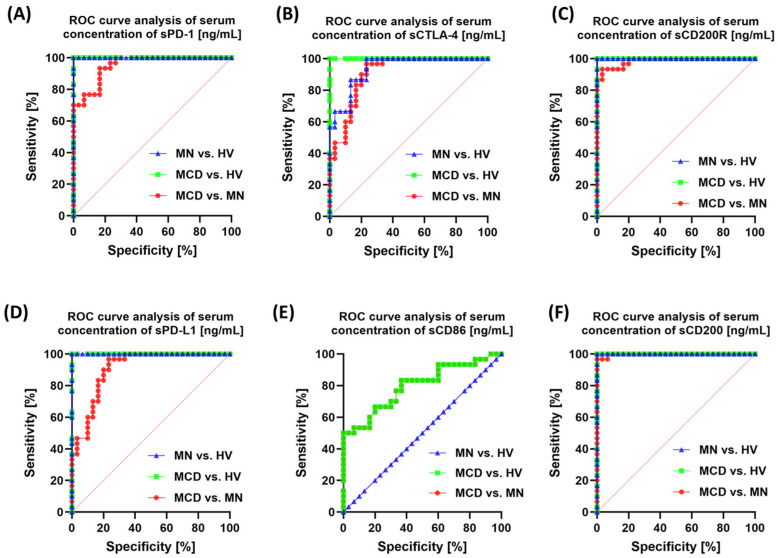
ROC curves for serum concentrations of soluble checkpoint molecules. Panels: (**A**) sPD-1, (**B**) sCTLA-4, (**C**) sCD200R, (**D**) sPD-L1, (**E**) sCD86, (**F**) sCD200. Colors: green—MCD vs. HV, blue—MN vs. HV, red—MCD vs. MN.

In serum, all soluble immune checkpoints and their ligands differentiated MCD and MN very well (sCD200 0.998; sCD200R 0.986; sPD-1 0.951; sPD-L1 0.908; sCTLA-4 0.908; all *p* < 0.0001), with sCD86 being weaker (0.799; *p* < 0.0001) and not distinguishing MN from HV (AUC 0.50) (Figure 13).

**Figure 14:**
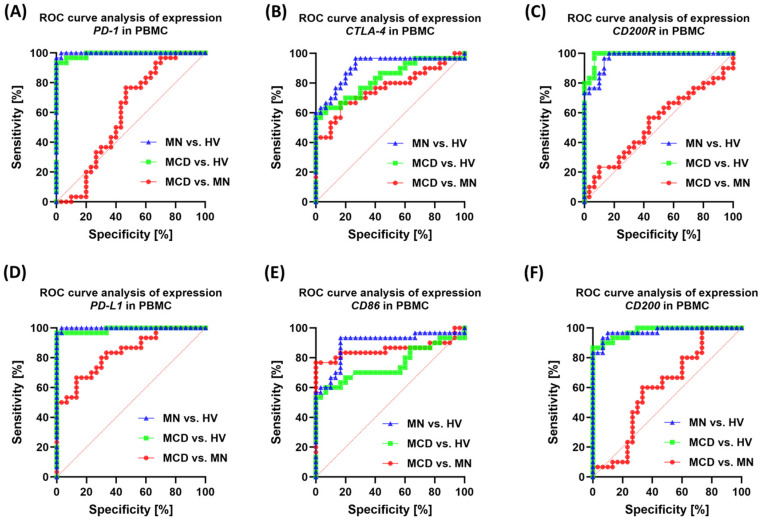
ROC curves for checkpoint gene expression in PBMC. Panels: (**A**) *PD-1*, (**B**) *CTLA-4*, (**C**) *CD200R*, (**D**) *PD-L1*, (**E**) *CD86*, (**F**) *CD200*. Curve colors: green—MCD vs. HV, blue—MN vs. HV, red—MCD vs. MN.

In PBMC, transcript expression best supported MCD vs. MN separation for *PD-L1* (0.84; *p* < 0.0001), *CD86* (0.86; *p* < 0.0001), and *CTLA-4* (0.76; *p* = 0.0004), whereas *PD-1*, *CD200*, and *CD200R* had low power (AUC ~0.53–0.60; *p* > 0.05) (Figure 14).

## 3. Discussion

### 3.1. Literature Analysis

Across the PubMed database, the term “glomerulonephritis” encompasses over 65,800 publications, reflecting the significant interest in the pathogenesis, classification, and treatment of glomerular diseases. However, when the search is narrowed to studies combining the terms “immune system” and “immune checkpoint,” only 28 entries remain. This small number of studies suggests that the role of immune checkpoints in the pathogenesis of glomerulopathies remains an underexplored area, with most data coming from translational studies in systemic lupus erythematosus, ANCA-related vasculitis, and observations of adverse effects of checkpoint inhibitors in oncology patients [50,51,52,53,54]. However, growing evidence suggests that immune checkpoints such as PD-1/PD-L1, CTLA-4/CD86, CD200/CD200R, and TIGIT play a key role in maintaining tissue tolerance in the kidney. Dysregulation of these checkpoints can lead to autoimmunity and glomerular damage, as demonstrated in studies of lupus nephritis [55,56] and idiopathic membranous nephropathy [56]. At the same time, experimental translational work has shown that PD-L1 overexpression in tubular epithelial cells may accelerate the development of interstitial fibrosis [42,57], and defects in regulatory pathways (e.g., NLRP12, P2RY8) result in loss of control of the interferon response and autoreactive B cell activation [55].

Given this limited yet rapidly expanding field of research, analyzing the expression of immune checkpoints in primary glomerulopathies—such as MCD and MN—represents an important step in understanding the mechanisms regulating the balance between activation and tolerance in the renal microenvironment. Incorporating this aspect into clinical-immunological studies may not only help differentiate between types of glomerular diseases, but could also open up prospects for checkpoint-based blood biomarkers and potential therapeutic targets aimed at modulating them.

In our study, which included newly diagnosed, untreated patients with MCD and MN, we analyzed the phenotype of peripheral blood cells, serum levels of soluble immune checkpoints, and the expression of their corresponding genes in PBMCs. At all three analytical levels, both diseases were clearly distinguishable from those of healthy volunteers.

When the three analytical layers were considered together (cell-surface phenotype, soluble serum forms, and PBMC transcript levels), several consistent and divergent patterns emerged. First, both MCD and MN showed a broadly concordant upregulation of the PD-1/PD-L1, CTLA-4/CD86 and CD200/CD200R axes across all three levels when compared with healthy volunteers, indicating a globally enhanced inhibitory checkpoint tone in primary glomerulopathies. Second, the relative magnitude of this upregulation differed between entities: MCD was characterized by higher PD-1 expression on CD4+ and CD8+ T cells and higher circulating concentrations of several soluble checkpoints (including sPD-1, sPD-L1, sCTLA-4, sCD200 and sCD200R), whereas MN showed relatively higher *PD-L1*, *CTLA-4* and *CD86* transcript levels in PBMC and a more pronounced PD-L1 expression on T and NK cells. Third, the CD200/CD200R axis was consistently increased at the cellular, soluble, and transcriptional levels in both diseases, without significant quantitative differences between MCD and MN at the transcript level, suggesting that this pathway represents a shared feature of checkpoint dysregulation in primary GN. Taken together, these patterns indicate a shared core of inhibitory checkpoint activation in MCD and MN, with superimposed, entity-specific quantitative shifts that may reflect differences in underlying immunopathology. However, the partially divergent trends between the cellular, soluble, and transcriptional layers should be interpreted with caution and viewed as hypothesis-generating, as differences may influence them in cellular sources, receptor shedding, turnover kinetics, and sampling time-points. These quantitative differences between MCD and MN are compatible with their known pathophysiological backgrounds: MN is an autoimmune disease associated with autoantibodies against podocyte antigens (PLA2R, THSD7A), whereas MCD is characterized by a predominant involvement of T lymphocytes and cytokines, mainly IL-13, leading to functional podocyte dysfunction without the formation of immune deposits [1,11,58,59,60,61,62].

At a more detailed level, the individual checkpoint axes showed distinct quantitative patterns between MCD and MN. The most pronounced differences concerned the PD-1/PD-L1 and CTLA-4/CD86 axes. In MCD, higher levels of PD-1 expression were observed on CD8+ and CD4+ lymphocytes, whereas PD-L1 expression on these cells was relatively lower. In MN, PD-L1 expression was more prominently increased on T and NK lymphocytes, while CD8+ T cells showed lower CTLA-4 expression [38,50,63,64,65].

The CD200/CD200R axis was strongly upregulated in both entities. In MCD, CD4+CD200R+ cells showed positive correlations with serum albumin, whereas in MN, lower CD200/CD200R levels were recorded in patients with higher proteinuria and lower serum albumin [4,42,54,55,65,66].

Correlation analysis revealed distinct patterns of associations between immunological markers and clinical parameters. In MCD, higher urea and creatinine concentrations were associated with lower levels of PD-1, PD-L1, and CTLA-4. In contrast, in MN, attenuation of the CD200/CD200R axis co-occurred with increased proteinuria and altered metabolic parameters. Opposite directions of correlation were also observed for three pairs of variables: CD4+CD200+ vs. CD8+CD200+, NK PD-L1+ vs. CD8+PD-L1+, and IgG vs. IgM—these correlations were positive in MCD and negative in MN [1,4,17,67,68].

In the biomarker analyses, the area under the ROC curve (AUC) for most markers reached values of 0.9 or higher in distinguishing patients from healthy individuals. For discrimination between MCD and MN, the highest AUC values were obtained for soluble sCD200 and sCD200R, as well as for the cellular phenotypes CD19+CD200R+, CD8+CD200+, CD4+PD-L1+, and CD8+CTLA-4+.

### 3.2. Limitations of the Study

Although the presented study results are promising, our study has several significant limitations. The analysis was performed once in newly diagnosed, untreated patients; we did not assess the dynamics of markers during the course of the disease or their changes in response to therapy. The sample size (MCD, MN, HV) is moderate, which limits the precision of estimation and the possibility of multivariate modeling (correction for confounding factors). A potential age/gender imbalance may influence specific immunological markers. In particular, patients with MN were significantly older than healthy volunteers, with a statistically significant age difference between these groups, which may confound direct comparisons of immune checkpoint profiles. We assessed peripheral blood (phenotype, serum, and PBMC expression) without direct validation in kidney biopsies (e.g., immunohistochemistry of PD-1/PD-L1, CTLA-4/CD86, and CD200/CD200R in glomeruli and interstitium). Therefore, direct conclusions about the architecture of the glomerular microenvironment cannot be drawn. Although we examined immunological profiles, the serological/antigenic status of MN (PLA2R, THSD7A, other antigens) was not correlated with checkpoint markers, thus preventing us from determining the relationship between the “autoantigen axis” and immune suppression. The single-center nature and possible ethnic/environmental differences limit the extrapolation of our results to other clinical populations (e.g., secondary forms of MN/MCD, cases after cancer immunotherapy). Further studies should be prospective, multicenter, and longitudinal, with serial sampling at disease onset, during treatment induction, in remission, and (if present) during relapse, to assess the dynamics of the PD-1/PD-L1, CTLA-4/CD86, and CD200/CD200R axes over time and their relationship to clinical activity and prognosis. Furthermore, these studies should also be expanded to include functional tests.

## 4. Materials and Methods

### 4.1. Participant Characteristics, Eligibility Criteria, and Study Material

A total of 90 participants were included in the study: 60 patients with glomerular kidney disease (GN) and 30 healthy volunteers (HV) serving as a reference group. Histopathological evaluation of renal biopsies enabled the identification of two equal cohorts within the GN group: 30 patients with minimal change disease (MCD) and 30 with membranous nephropathy (MN). The MCD patients comprised 15 women and 15 men, while the MN cohort consisted of 12 women and 18 men (total: 27 women and 33 men). The HV group consisted of 13 women and 17 men; the volunteers were age-matched and did not report any chronic systemic diseases.

Inclusion criteria included: age ≥ 18 years, recent diagnosis of primary GN confirmed by renal biopsy, no prior immunosuppression or steroid therapy, no active infection on the day of sample collection, and written informed consent. Specifically, all HV were ≥18 years of age, had no history of kidney disease, autoimmune disorders, or arterial hypertension, had no prior immunosuppressive or steroid therapy, and had no active infection on the day of sampling. They also provided written informed consent. Exclusion criteria included: secondary forms of glomerulopathy (e.g., lupus nephropathy, amyloidosis), HIV, HBV, or HCV infection, systemic autoimmune diseases, organ transplantation, known hematological or neoplastic diseases, and use of immunomodulatory medications or antibiotics within the last 3 months for both patients and HV. In addition, individuals with obesity (BMI ≥ 30 kg/m^2^) were not eligible, and no obese subjects were included in the HV group.

The biological material consisted of venous blood collected from the basilic vein. From each participant, 10 mL of blood was collected in EDTA tubes and 5 mL in tubes without anticoagulant to obtain serum for serological testing. Samples were processed immediately after collection, following procedures to ensure cell viability and protein stability. All activities were carried out in accordance with the principles of good laboratory practice and bioethical requirements.

### 4.2. Staining and Acquisition Parameters for Flow Cytometry

Lymphocyte phenotype profiling was performed using multiparameter flow cytometry with a panel of antibodies targeting lineage markers (CD3, CD4, CD8, CD16, CD19, CD56) and regulatory molecules (PD-1, PD-L1, CTLA-4, CD86, CD200, CD200R). The panel composition (clones, fluorochromes, catalog numbers, and suppliers) is summarized in Table S9 (Supplementary Materials). BD Horizon™ Brilliant Stain Buffer Plus was used to minimize emission overlap; staining was performed for 20 min at room temperature in the dark, then washed in BD Pharmingen™ Stain Buffer (FBS) (BD Biosciences, San Jose, CA, USA). Intracellular staining was performed using the BD Cytofix/Cytoperm™ kit (BD Biosciences, San Jose, CA, USA). Acquisition was performed on a CytoFLEX LX cytometer (Beckman Coulter, Brea, CA, USA), and analysis was conducted in Kaluza v2.1. Compensation and quality control were performed using the VersaComp Antibody Capture Kit (Beckman Coulter, Brea, CA, USA) and CytoFLEX Daily QC Fluorospheres (Beckman Coulter, Brea, CA, USA), respectively. For each sample, at least 15,000 events were recorded, gated based on FSC–SSC parameters for lymphocytes and then on CD45 expression. Positivity thresholds for checkpoint molecules were established using appropriate FMO controls and applied consistently across samples. In this study, the primary analysis parameter was the percentage of cells expressing a given molecule within defined subpopulations (e.g., % PD-1^+^ among CD4^+^, CD8^+^, CD19^+^, or NK cells). All flow cytometry results were expressed as the percentage of positive cells in the parent population, and comparisons between the analyzed groups were made based on this percentage. The schematic gating strategy is shown in Figure S2.

### 4.3. Isolation of Peripheral Blood Mononuclear Cells (PBMC)

PBMC were obtained from whole blood collected in EDTA tubes. Isolation was performed by density gradient separation using Gradisol L (Aqua-Med, Łódź, Poland; density 1.077 g/mL), according to the manufacturer’s instructions and standards described in the literature. Blood was diluted 1:1 with Ca^2+^/Mg^2^-free PBS buffer and gently applied to the separation medium in 15 mL tubes. Samples were then centrifuged for 20 min at 700× *g* at room temperature, without braking the rotor. After centrifugation, the PBMC-rich buffy coat was collected with a sterile pipette and washed twice in PBS (10 min, 400× *g*, 4 °C) to remove residual gradient medium and cellular debris.

### 4.4. RNA Extraction from PBMCs, cDNA Synthesis, and Expression Analysis by qPCR

RNA isolation. Total RNA was extracted from PBMCs using the Total RNA Mini Plus kit (A&A Biotechnology, Gdańsk, Poland) according to the instructions. The obtained material was eluted in 30 µL of RNase-free water. Quantity and purity were assessed spectrophotometrically (BioSpec-nano, Shimadzu Corporation, Kyoto, Japan), using an A260/A280 ≥ 1.8 as the eligibility criterion. Reverse transcription was performed using 500 ng of RNA and the iScript™ cDNA Synthesis Kit (Bio-Rad, Hercules, CA, USA) in a volume of 20 µL. Program: 25 °C/5 min (priming), 42 °C/30 min (synthesis), 85 °C/5 min (inactivation). cDNA was stored at −80 °C. qPCR. Expression of PD-1, PD-L1, CTLA-4, CD86, CD200, and CD200R was determined using SYBR Green and SsoAdvanced™ Universal SYBR^®^ Green Supermix (Bio-Rad) and primer pairs specific for the studied transcripts. Reaction composition: 20 µL: 10 µL Supermix, 1 µL primer mix, 2 µL cDNA, 7 µL nuclease-free water. Conditions on CFX96 (Bio-Rad): initial denaturation 95 °C/30 s, then 40 cycles: 95 °C/5 s, 60 °C/30 s. Results were normalized to ACTB (β-actin) and calculated by the 2^^−ΔΔCt^ method.

### 4.5. Serum Checkpoint Analysis (ELISA)

Soluble PD-1, PD-L1, CTLA-4, CD86, CD200, and CD200R concentrations were determined using a sandwich ELISA assay with ENLIBIO kits (Wuhan, China). Absorbance was recorded on a Victor3 (PerkinElmer, Waltham, MA, USA). Results were reported as pg/mL or ng/mL according to the manufacturer’s specifications.

### 4.6. Statistical Analysis

Calculations were performed in GraphPad Prism v9.0 (GraphPad Software, San Diego, CA, USA). Normality of distributions was verified using the Shapiro–Wilk test. Due to the lack of normality for most variables, nonparametric tests were used: the Mann–Whitney test for comparisons between two independent groups and the Kruskal–Wallis test for multigroup analyses (MN, MCD, and HV subgroups). In the event of significance in the overall analysis (*p* < 0.05), post hoc comparisons were performed using Dunn’s test. The level of significance was set at *p* < 0.05. Data were presented as median [Q1–Q3]. To assess the diagnostic properties of the studied parameters (including serum checkpoint concentrations), ROC analysis was performed. The AUC was calculated along with its 95% CI and *p*-value to distinguish patients with glomerulopathies from healthy individuals and to differentiate disease subtypes.

## 5. Conclusions

This study provided new insights into the immunoregulatory mechanisms of primary glomerulopathies, demonstrating that both minimal change disease (MCD) and membranous nephropathy (MN) share a common immune imbalance but differ in the profile of specific checkpoint pathways. MCD exhibited a dominant pattern of T cell activation with predominant expression of PD-1 and CTLA-4, while MN exhibited a predominance of humoral activation and PD-L1 expression with a weakened CD200/CD200R axis. These results confirm that the mechanisms of maintaining immune tolerance in glomerular diseases are complex and vary depending on the dominant component of the immune response. These differences in the expression profiles and immunological correlations between MCD and MN may have clinical significance, particularly in the context of identifying biomarkers with diagnostic and prognostic potential. The use of soluble forms of checkpoints, such as sPD-1, sPD-L1, sCD200, and sCD200R, may be a promising, non-invasive tool for assessing disease activity and differentiating between types of glomerulopathy. Future research should focus on longitudinal analyses that encompass the dynamics of checkpoint expression changes during treatment and disease remission. It is also necessary to expand the study to include an analysis of renal tissue biopsies, which will enable a better understanding of the inflammatory microenvironment and its impact on the course of glomerulopathy. The use of modern methods, such as single-cell RNA-seq analysis, will enable a more precise definition of the cell populations involved in pathogenesis and their interactions through checkpoint axes. The long-term goal should be to develop personalized immunomodulatory strategies that precisely modulate the PD-1/PD-L1, CTLA-4/CD86, and CD200/CD200R pathways, thereby supporting immunosuppressive therapy and reducing the risk of disease relapse. Integrating immunological knowledge with clinical practice offers a realistic opportunity to introduce modern, targeted treatments for primary glomerular diseases.

## Figures and Tables

**Table 1:** Demographic and laboratory characteristics of newly diagnosed, untreated patients with minimal change disease (MCD) and membranous nephropathy (MN) and healthy volunteers (HV).

Parameters	MCD Median (Q1–Q3)	MN Median (Q1–Q3)	HV Median (Q1–Q3)	*p*-Value			
				All	MCD vs. MN	MCD vs. HV	MN vs. HV
Age	49.50 (42.75–23.00)	57.50 (49.00–34.00)	44.50 (40.25–35.00)	0.0034	0.0711	0.9077	0.003
WBC [10^3^/mm^3^]	6.65 (5.83–4.33)	6.50 (5.40–4.33)	6.30 (5.91–4.20)	0.5864	>0.9999	0.9045	>0.9999
NEU [10^3^/mm^3^]	5.35 (5.01–3.90)	4.17 (3.70–2.75)	6.65 (4.82–3.06)	<0.0001	0.0007	>0.9999	<0.0001
MON [10^3^/mm^3^]	0.48 (0.40–0.13)	0.69 (0.56–0.26)	0.57 (0.43–0.20)	0.0003	0.0002	0.1313	0.1268
LYM [10^3^/mm^3^]	2.08 (1.83–0.42)	1.92 (1.37–0.27)	1.95 (1.84–1.20)	0.3432	0.4364	>0.9999	>0.9999
EOS [10^3^/mm^3^]	0.20 (0.18–0.09)	0.17 (0.14–0.00)	0.17 (0.12–0.00)	0.0059	0.0389	0.0083	>0.9999
BAS [10^3^/mm^3^]	0.03 (0.01–0.00)	0.02 (0.00–0.00)	0.02 (0.00–0.00)	0.122	0.1888	0.2794	>0.9999
RBC [10^6^/mm^3^]	4.24 (3.87–3.20)	4.11 (3.68–3.10)	4.51 (4.24–3.67)	0.0108	>0.9999	0.0331	0.0231
HGB [g/dL]	12.24 (11.10–9.72)	12.90 (11.14–9.20)	14.76 (13.03–10.70)	0.0003	>0.9999	0.0005	0.0067
PLT [10^3^/mm^3^]	238.46 (196.86–120.00)	227.00 (193.50–126.42)	247.00 (212.54–188.00)	0.1037	>0.9999	0.2368	0.1646
Urea [mg/dL]	56.22 (34.99–17.79)	30.78 (23.32–15.48)	21.00 (18.80–14.00)	<0.0001	0.0342	<0.0001	0.003
Creatine [mg/dL]	1.04 (0.77–0.37)	0.85 (0.67–0.38)	0.70 (0.70–0.56)	0.0119	0.5901	0.009	0.2799
Uric acid [mg/dL]	7.38 (6.03–3.80)	5.80 (4.99–3.70)	4.20 (3.60–3.29)	<0.0001	0.0288	<0.0001	<0.0001
eGFR	64.52 (61.26–30.30)	87.32 (76.76–35.00)	129.00 (126.00–117.50)	<0.0001	0.0249	<0.0001	<0.0001
Cholesterol [mg/dL]	279.25 (246.53–139.67)	259.00 (209.83–118.68)	149.20 (134.25–112.80)	<0.0001	0.6923	<0.0001	<0.0001
Triglycerides [mg/dL]	170.38 (150.28–51.63)	124.32 (104.78–75.44)	115.00 (69.75–55.46)	<0.0001	0.0913	<0.0001	0.033
HDL [mg/dL]	61.17 (49.23–34.00)	59.14 (48.74–25.67)	60.00 (50.00–38.00)	0.7384	>0.9999	>0.9999	>0.9999
LDL [mg/dL]	199.86 (177.68–72.48)	153.03 (110.71–60.95)	102.00 (93.00–79.00)	<0.0001	0.0717	<0.0001	0.0036
IgG [g/L]	4.20 (2.91–2.00)	4.93 (4.25–1.91)	5.20 (4.87–2.09)	0.0025	0.0493	0.0023	>0.9999
IgM [g/L]	1.06 (0.87–0.30)	1.00 (0.54–0.34)	1.80 (1.13–0.70)	0.0002	>0.9999	0.003	0.0003
IgA [g/L]	2.06 (1.73–0.82)	2.21 (1.56–0.75)	2.42 (1.82–0.95)	0.3194	>0.9999	0.3951	>0.9999
Total protein [g/dL]	4.80 (4.03–2.83)	4.48 (3.95–3.10)	7.40 (7.09–3.60)	<0.0001	>0.9999	<0.0001	<0.0001
Albumin [g/L]	1.97 (1.68–0.80)	2.48 (1.95–1.20)	4.28 (3.99–2.33)	<0.0001	0.5633	<0.0001	<0.0001
Proteinuria [g/24 h]	5.50 (4.13–2.49)	5.87 (3.88–1.00)	0.00 (0.00–0.00)	<0.0001	>0.9999	<0.0001	<0.0001

Abbreviation: BAS: basophils, eGFR: estimated glomerular filtration rate, EOS: eosinophils, HGB: hemoglobin, HDL: high-density lipoprotein cholesterol, HV: healthy volunteers, IgA: immunoglobulin A, IgG: immunoglobulin G, IgM: immunoglobulin M, LDL: low-density lipoprotein cholesterol, LYM: lymphocytes, MCD: minimal change disease, MN: membranous nephropathy, MON: monocytes, NEU: neutrophils, PLT: platelets, RBC: red blood cells, WBC: white blood cells

**Table 2:** Basic peripheral blood immunophenotype (cell percentages) in patients with minimal change disease (MCD), membranous nephropathy (MN), and healthy volunteers (HV).

Parameters	MCD Median (Q1–Q3)	MN Median (Q1–Q3)	HV Median (Q1–Q3)	*p*-Value			
				ALL	MCD vs. MN	MCD vs. HV	MN vs. HV
CD45+ [%]	97.81 (97.17–99.11)	97.92 (95.81–98.48)	98.65 (97.09–99.33)	0.3101	>0.9999	>0.9999	0.3782
CD3+ [%]	70.85 (66.88–76.30)	72.75 (66.39–78.60)	73.16 (70.47–78.95)	0.2793	>0.9999	0.3311	>0.9999
CD19+ [%]	10.91 (8.40–16.42)	11.12 (9.17–12.60)	12.36 (7.97–14.74)	0.9329	>0.9999	>0.9999	>0.9999
CD3-CD16+CD56+ [%]	11.65 (6.18–18.71)	8.60 (6.93–15.19)	9.96 (8.63–14.06)	0.3557	0.9688	>0.9999	0.4858
CD4+ [%]	41.07 (33.27–48.50)	42.11 (34.88–48.33)	42.31 (36.01–49.01)	0.8298	>0.9999	>0.9999	>0.9999
CD8+ [%]	25.79 (22.35–29.73)	25.75 (22.25–30.62)	27.11 (22.17–29.98)	0.6393	>0.9999	>0.9999	>0.9999
Ratio CD4/CD8	1.58 (1.02–2.14)	1.51 (1.35–2.07)	1.58 (0.97–2.27)	0.9431	>0.9999	>0.9999	>0.9999

## Data Availability

The original contributions presented in this study are included in the article/Supplementary Material. Further inquiries can be directed to the corresponding author.

## Supplementary Materials

The following supporting information can be downloaded at: https://www.mdpi.com/article/10.3390/ijms262311371/s1.

## Abbreviations

Abbreviation	Full Term
MN	Membranous nephropathy
MCD	Minimal Change Disease
HV	Healthy volunteers
PD-1	Programmed cell death protein 1
PD-L1	Programmed death-ligand 1
CTLA-4	Cytotoxic T-lymphocyte-associated protein 4
CD86	Cluster of differentiation 86
CD200R	CD200 receptor
CD200	Cluster of differentiation 200
PBMC	Peripheral blood mononuclear cells
ROC	Receiver operating characteristic
sPD-1	Soluble PD-1
sPD-L1	Soluble PD-L1
sCTLA-4	Soluble CTLA-4
sCD86	Soluble CD86
sCD200	Soluble CD200
sCD200R	Soluble CD200R
GN	Glomerulonephritis
NK	Natural killer (cells)
ELISA	Enzyme-linked immunosorbent assay
CD4+/CD8+ T cells	T lymphocyte subsets expressing CD4 or CD8
CD19+ B cells	B lymphocytes expressing CD19
