# Supplementary material for: Immune Checkpoint Signatures in Minimal Change Disease and Membranous Nephropathy: Divergent Pathways of a Shared Imbalance
Source: Int J Mol Sci. 2025 Nov 25;26(23):11371. doi: 10.3390/ijms262311371 (PMC12692590; doi:10.3390/ijms262311371)
Supplement: Supplementary file 1 [file ijms-26-11371-s001.zip › ijms-3964904-supplementary.pdf]

Supplemntary Materials Table S1. Percentage of cells positive for checkpoint molecules in the peripheral blood of newly diagnosed, untreated patients with minimal change disease (MCD) and membranous nephropathy (MN) and healthy volunteers (HV).

	MCD	MN	HV	ALL	MCD vs. MN	MCD vs. HV	MN vs. HV
CD4+PD-1+ [%]	12.07 (7.07-17.19)	8.90 (8.04-9.77)	0.87 (0.59-1.97)	<0.0001	0.7548	<0.0001	<0.0001
CD8+PD-1+[%]	18.96 (10.75-21.23)	10.00 (8.52-12.41)	1.56 (0.96-2.31)	<0.0001	0.0815	<0.0001	<0,.0001
CD19+PD-1+[%]	2.25 (1.59-3.74)	2.43 (1.45-3.38)	0.25 (0.11-0.62)	<0.0001	>0.9999	<0.0001	<0.0001
CD3-CD16+CD56+PD-1+[%]	5.77 (5.18-7.88)	5.48 (3.68-7.89)	2.89 (2.48-3.26)	<0.0001	>0.9999	<0.0001	<0.0001
CD4+PD-L1+[%]	6.41 (4.54-8.93)	15.34 (11.57-22.47)	1.12 (0.76-1.52)	<0.0001	0.0002	<0.0001	<0.0001
CD8+PD-L1+[%]	5.75 (2.71-8.49)	4.31 (1.49-5.31)	0.47 (0.31-0.64)	<0.0001	0.1874	<0.0001	<0.0001
CD19+PD-L1+[%]	3.97 (2.59-5.44)	3.67 (2.37-5.19)	0.59 (0.37-0.95)	<0.0001	>0.9999	<0.0001	<0.0001
CD3-CD16+CD56+PD-L1+[%]	4.63 (3.81-5.44)	2.81 (1.41-4.57)	3.53 (2.60-4.19)	<0.0001	0.0002	0.0017	>0.9999
CD4+CTLA-4+[%]	3.16 (1.87-4.92)	5.10 (3.16-8.06)	7.16 (6.55-7.67)	<0.0001	0.0072	<0.0001	0.023
CD8+CTLA-4+[%]	5.88 (4.40-7.36)	2.74 (1.80-3.90)	5.78 (5.48-6.45)	<0.0001	<0.0001	>0.9999	<0.0001
CD19+CTLA-4+[%]	5.46 (3.79-6.72)	8.17 (6.77-9.22)	7.34 (6.43-7.91)	<0.0001	<0.0001	0.018	0.1628
CD3-CD16+CD56+CTLA-4+[%]	1.46 (0.89-2.37)	1.84 (0.89-2.48)	0.73 (0.30-1.44)	0.0006	>0.9999	0.0057	0.0013
CD4+CD86+[%]	1.48 (0.93-2.09)	2.04 (1.62-2.69)	3.47 (1.93-4.02)	<0.0001	0.0924	<0.0001	0.0594
CD8+CD86+[%]	1.51 (0.75-2.57)	2.25 (1.80-2.65)	3.21 (2.03-3.65)	0.0002	0.1502	0.0001	0.0907
CD19+CD86+[%]	2.28 (1.47-2.54)	1.82 (1.53-2.57)	3.96 (3.01-4.70)	<0.0001	>0.9999	<0.0001	<0.0001
CD3-CD16+CD56+CD86+[%]	3.02 (1.76-4.14)	1.13 (0.78-2.06)	0.74 (0.34-1.32)	<0.0001	<0.0001	<0.0001	0.1282

CD4+CD200+[%]	44.52 (39.66-49.09)	44.93 (37.46-51.87)	4.76 (4.54-5.08)	<0.0001	>0.9999	<0.0001	<0.0001
CD8+CD200+[%]	38.70 (35.41-41.71)	29.40 (21.42-34.85)	5.15 (4.48-5.46)	<0.0001	0.0008	<0.0001	<0.0001
CD19+CD200+[%]	35.96 (32.01-44.19)	47.63 (38.41-53.52)	5.08 (4.62-5.25)	<0.0001	0.0755	<0.0001	<0.0001
CD3-CD16+CD56+CD200+[%]	7.17 (5.79-8.70)	6.20 (4.68-8.19)	5.67 (4.80-6.30)	0.0024	0.2069	0.0015	0.2935
CD4+CD200R+[%]	48.22 (44.82-55.74)	62.47 (55.17-72.19)	7.30 (5.49-8.41)	<0.0001	0.0068	<0.0001	<0.0001
CD8+CD200R+[%]	49.47 (46.71-55.11)	48.33 (43.10-52.51)	7.68 (5.85-8.95)	<0.0001	>0.9999	<0.0001	<0.0001
CD19+CD200R+[%]	36.67 (34.52-39.85)	47.22 (42.86-49.85)	27.46 (18.22-35.00)	<0.0001	<0.0001	0.0071	<0.0001
CD3-CD16+CD56+CD200R+[%]	9.58 (7.06-12.48)	7.08 (6.02-8.31)	6.40 (5.44-7.30)	<0.0001	0.0206	<0.0001	0.1468

Supplementary Materials Table S2. Serum concentrations of soluble immune checkpoint molecules (sPD-1, sPD-L1, sCTLA-4, sCD86, sCD200, and sCD200R) in patients with minimal change disease (MCD), membranous nephropathy (MN), and healthy volunteers (HV).

Parameters	MCD Median (Q1-Q3)	MN Median (Q1-Q3)	HV Median (Q1-Q3)	p-Value			
				ALL	MCD vs. MN	MCD vs. HV	MN vs. HV
sPD-1 [ng/mL]	18.71 (14.72-19.77)	12.25 (10.30-13.33)	1.79 (1.66-1.85)	<0.0001	0.0009	<0.0001	<0.0001
sPD-L1 [ng/mL]	11.60 (9.13-12.26)	7.60 (6.39-8.27)	4.68 (4.41-5.07)	<0.0001	0.0003	<0.0001	<0.0001
sCTLA-4 [ng/mL]	4.67 (4.34-5.52)	6.00 (5.31-6.47)	6.00 (5.31-6.47)	<0.0001	0.0002	0.0002	>0.9999

sCD86 [ng/mL]	20.16 (18.98-2.36)	13.46 (12.50-14.19)	4.07 (3.84-4.41)	<0.0001	<0.0001	<0.0001	<0.0001
sCD200 [ng/mL]	19.81 (19.03-22.17)	13.40 (12.32-14.20)	3.60 (3.40-3.90)	<0.0001	<0.0001	<0.0001	<0.0001
sCD200R [ng/mL]	18.71 (14.72-19.77)	12.25 (10.30-13.33)	1.79 (1.66-1.85)	<0.0001	0.0009	<0.0001	<0.0001

Supplementary Materials Table S3. Expression levels of immune checkpoint genes (qPD-L1, qCTLA-4, qCD86, qCD200, qCD200R, and qPD-1) in peripheral blood mononuclear cells (PBMCs) from patients with minimal change disease (MCD), membranous nephropathy (MN), and healthy volunteers (HV).

Parameters	MCD Median (Q1-Q3)	MN Median (Q1-Q3)	HV Median (Q1-Q3)	p-Value			
				ALL	MCD vs. MN	MCD vs. HV	MN vs. HV
qPD-L1	44.71 (21.93-57.71)	28.63 (21.53-39.60)	0.74 (0.40-3.57)	<0.0001	>0.9999	<0.0001	<0.0001
qCTLA-4	12.10 (9.36-15.12)	19.80 (14.64-27.59)	0.81 (0.52-1.79)	<0.0001	0.0074	<0.0001	<0.0001
qCD86	3.79 (2.30-4.79)	5.53 (4.28-6.82)	0.92 (0.43-2.50)	<0.0001	0.0212	0.0011	<0.0001
qCD200	2.62 (1.93-4.13)	8.11 (5.30-10.21)	0.80 (0.40-2.97)	<0.0001	0.0005	0.0461	<0.0001
qCD200R	14.29 (8.63-19.59)	11.39 (8.68-15.62)	1.00 (0.48-2.20)	<0.0001	>0.9999	<0.0001	<0.0001
qPD-L1	16.48 11.04-21.55)	17.36 (11.24-22.85)	1.11 (0.50-2.39)	<0.0001	>0.9999	<0.0001	<0.0001

Supplementary Material Table S4. Comparison of clinical, hematological, biochemical, and immunological parameters between female and male patients with minimal change disease (MCD), membranous nephropathy (MN), and healthy volunteers (HV).

	MCD Female	MCD Male	MN Female	MN Male	HV Female	HV Male	ALL	MCD Female vs. MCD Male	MCD Female vs. MN Female	MCD Female vs. MN Male	MCD Female vs. HV Female	MCD Female vs. HV Male	MCD Male vs. MN Female	MCD Male vs. MN Male	MCD Male vs. HV Female	MCD Male vs. HV Male	MN Female vs. MN Male	MN Female vs. HV Female	MN Female vs. HV Male	MN Male vs. HV Female	MN Male vs. HV Male	HV Female vs. HV Male
Age	48.00 (45.00-59.50)	50.00 (32.50- 57.00	53.50 (46.50-57.50)	63.50 (51.00-70.75)	41.00 (39.00-44.00)	48.00 (44.00-55.00)	0.0036	>0.9999	>0.9999	0.5452	0.7522	>0.9999	>0.9999	0.1411	>0.9999	>0.9999	>0.9999	0.4069	>0.9999	0.0008	0.294	0.9471

PLT	HGB	RBC	BAS	EOS	LYM	MON	NEU	WBC
240.00 (203.32-260.35)	12.00 (11.00-12.43)	4.24 (3.96-4.35)	0.02 (0.00-0.10)	0.20 (0.20-0.28)	2.00 (1.8-2.25)	0.47 (0.40-0.52)	5.20 (4.93-5.75)	6.36 (6.20-7.20)
236.91 (192.61-268.81)	13.30 (11.68-14.05)	4.33 (3.90-4.65)	0.04 (0.01-0.09)	0.22 (0.15-0.29)	2.21 (1.84-2.67)	0.49 (0.38-0.58)	5.60 (5.07-6.00)	6.98 (5.40-9.13)
245.26 (194.36-280.77)	11.05 (10.69-12.19)	3.81 (3.50-4.06)	0.01 (0.00-0.02)	0.18 (0.10-0.21)	1.58 (1.18-2.41)	0.61 (0.48-0.78)	3.71 (3.19-4.08)	5.65 (5.04-6.53)
218.14 (195.59-243.75)	13.55 (13.03-15.13)	4.51 (4.02-4.98)	0.02 (0.01-0.03)	0.17 (0.14-0.19)	1.95 (1.71-2.26)	0.78 (0.66-0.81)	4.60 (4.07-4.84)	6.97 (6.08-8.07)
254.00 (245.00-308.00)	14.90 (14.00-15.00)	4.51 (4.45-4.79)	0.02 (0.00-0.02)	0.16 (0.13-0.18)	1.97 (1.90-2.50)	0.50 (0.40-0.56)	6.90 (6.80-7.25)	6.30 (6.00-6.80)
231.00 (209.62-282.00)	13.10 (12.78-15.70)	4.40 (4.10-4.93)	0.02 (0.01-0.02)	0.17 (0.10-0.20)	1.92 (1.50-2.10)	0.65 (0.50-0.79)	5.90 (4.70-6.70)	6.30 (5.90-6.80)
0.2606	<0.0001	0.0006	0.1675	0.0583	0.3205	0.0002	<0.0001	0.5224
>0.9999	>0.9999	>0.9999	>0.9999	>0.9999	>0.9999	>0.9999	>0.9999	>0.9999
>0.9999	>0.9999	>0.9999	0.3711	0.9968	>0.9999	>0.9999	0.0799	>0.9999
>0.9999	0.0687	>0.9999	>0.9999	0.5296	>0.9999	0.002	0.6641	>0.9999
>0.9999	0.0008	0.1796	>0.9999	0.1852	>0.9999	>0.9999	>0.9999	>0.9999
>0.9999	0.0334	>0.9999	>0.9999	0.3913	>0.9999	0.1823	>0.9999	>0.9999
>0.9999	0.3865	0.3302	0.2419	>0.9999	0.5436	>0.9999	0.0164	>0.9999
>0.9999	>0.9999	>0.9999	>0.9999	>0.9999	>0.9999	0.0035	0.1745	>0.9999
0.5321	0.207	0.6597	>0.9999	0.5954	>0.9999	>0.9999	>0.9999	>0.9999
>0.9999	>0.9999	>0.9999	>0.9999	>0.9999	>0.9999	0.269	>0.9999	>0.9999
>0.9999	0.0111	0.0101	0.8999	>0.9999	>0.9999	>0.9999	>0.9999	>0.9999
>0.9999	0.0001	0.0006	>0.9999	>0.9999	0.859	>0.9999	0.0003	>0.9999
>0.9999	0.0052	0.0089	>0.9999	>0.9999	>0.9999	>0.9999	0.0301	>0.9999
0.3557	>0.9999	>0.9999	>0.9999	>0.9999	>0.9999	0.01	0.0045	>0.9999
>0.9999	>0.9999	>0.9999	>0.9999	>0.9999	>0.9999	>0.9999	0.3071	>0.9999
>0.9999	>0.9999	>0.9999	>0.9999	>0.9999	>0.9999	0.4766	>0.9999	>0.9999

IgG	LDL	HDL	Trójglicerydy	Cholesterol	eGFR	Uric acid	Creatine	Urea
4.33 (3.81-4.85)	204.00 (186.08-211.00)	61.44 (55.96-66.60)	176.80 (156.89-194.50)	281.49 (240.63-318.95)	66.65 (61.66-70.20)	8.20 (7.16-8.81)	1.41 (0.86-1.87)	68.95 (41.99-90.15)
3.21 (2.51-4.62)	185.64 (145.71-222.65)	53.84 (44.08-71.12)	168.76 (147.46-192.48)	272.00 (247.30-339.00)	62.06 (60.35-69.65)	6.98 (5.62-7.65)	0.85 (0.77-1.19)	43.62 (35.38-67.60)
4.82 (4.10-5.56)	163.15 (120.53-209.10)	60.50 (50.31-67.54)	116.77 (96.08-124.31)	272.62 (198.38-303.31)	83.23 (77.19-88.89)	5.12 (4.76-5.88)	0.69 (0.55-0.99)	22.89 (20.90-54.21)
5.04 (4.54-5.86)	149.41 (110.71-176.56)	55.64 (48.74-67.80)	152.76 (121.25-204.50)	259.00 (213.30-281.25)	89.00 (77.62-93.50)	6.10 (5.16-6.35)	0.96 (0.81-1.16)	31.95 (27.44-50.47)
5.12 (4.83-5.50)	104.00 (97.00-112.80)	55.00 (50.00-64.00)	133.00 (120.00-146.00)	147.00 (133.00-157.00)	129.00 (126.00-137.00)	3.95 (3.60-4.30)	0.75 (0.70-0.80)	25.00 (18.80-27.00)
5.52 (4.89-6.49)	102.00 (90.00-120.00)	64.00 (51.70-73.00)	79.00 (69.00-120.00)	157.00 (139.00-169.00)	134.42 (126.00-137.00)	4.20 (3.60-4.80)	0.70 (0.70-0.80)	20.00 (18.80-24.00)
0.0119	<0.0001	0.7821	<0.0001	<0.0001	<0.0001	<0.0001	0.0012	<0.0001
>0.9999	>0.9999	>0.9999	>0.9999	>0.9999	>0.9999	>0.9999	>0.9999	>0.9999
>0.9999	>0.9999	>0.9999	0.0793	>0.9999	>0.9999	0.0802	0.0326	0.1196
>0.9999	0.3765	>0.9999	>0.9999	>0.9999	>0.9999	0.6957	>0.9999	>0.9999
>0.9999	0.0007	>0.9999	0.2967	<0.0001	<0.0001	<0.0001	0.0321	0.0025
0.363	0.0001	>0.9999	0.0002	0.0001	<0.0001	<0.0001	0.0108	<0.0001
0.7652	>0.9999	>0.9999	0.1316	>0.9999	0.9621	0.9589	>0.9999	0.2354
0.1431	>0.9999	>0.9999	>0.9999	>0.9999	0.3936	>0.9999	>0.9999	>0.9999
0.2853	0.0165	>0.9999	0.4631	0.0001	<0.0001	0.0001	>0.9999	0.0064
0.0062	0.0041	>0.9999	0.0006	0.0004	<0.0001	0.0003	0.7102	0.0002
>0.9999	>0.9999	>0.9999	0.4327	>0.9999	>0.9999	>0.9999	0.4024	>0.9999
>0.9999	0.425	>0.9999	>0.9999	0.0056	0.0075	0.222	>0.9999	>0.9999
>0.9999	0.2032	>0.9999	>0.9999	0.0156	0.0021	0.5212	>0.9999	>0.9999
>0.9999	0.5609	>0.9999	>0.9999	0.0016	0.0037	0.0029	0.4163	0.1396
>0.9999	0.2477	>0.9999	0.0029	0.0045	0.0007	0.0074	0.2079	0.0091
>0.9999	>0.9999	>0.9999	>0.9999	>0.9999	>0.9999	>0.9999	>0.9999	>0.9999

Proteinuria	Albumin	Total protein	IgA	IgM
5.81 (4.26-6.39)	1.94 (1.73-2.70)	5.10 (4.83-5.49)	1.94 (1.73-2.15)	1.15 (0.98-1.25)
4.80 (3.79-6.65)	2.00 (1.60-2.35)	4.60 (3.56-4.74)	2.30 (1.76-2.65)	0.94 (0.77-1.15)
4.17 (3.55-5.14)	2.41 (2.05-2.74)	4.34 (3.74-4.93)	2.04 (1.57-2.57)	1.05 (0.77-1.42)
6.93 (5.49-8.00)	2.48 (1.96-2.92)	4.57 (4.13-5.18)	2.44 (1.67-3.01)	0.92 (0.50-1.38)
0.00 (0.00-0.00)	4.28 (4.18-4.40)	7.80 (7.24-7.88)	2.30 (1.80-2.60)	1.50 (1.20-2.16)
0.00 (0.00-0.00)	4.26 (3.70-4.49)	7.35 (6.99-7.60)	2.44 (1.87-3.00)	1.80 (1.10-2.30)
<0.0001	<0.0001	<0.0001	0.263	0.0026
>0.9999	>0.9999	0.7366	>0.9999	>0.9999
>0.9999	>0.9999	>0.9999	>0.9999	>0.9999
>0.9999	>0.9999	>0.9999	>0.9999	>0.9999
<0.0001	<0.0001	0.0234	>0.9999	>0.9999
<0.0001	0.0001	0.0584	0.5876	0.5282
>0.9999	>0.9999	>0.9999	>0.9999	>0.9999
>0.9999	>0.9999	>0.9999	>0.9999	>0.9999
0.0002	<0.0001	<0.0001	>0.9999	0.1945
<0.0001	<0.0001	<0.0001	>0.9999	0.0489
>0.9999	>0.9999	>0.9999	>0.9999	>0,9999
0.0028	0.0027	0.0001	>0.9999	0.5617
0.0011	0.0052	0.0003	>0.9999	0.199
<0.0001	0.0006	0.0002	>0.9999	0.0831
<0.0001	0.0011	0.0004	>0.9999	0.0154
>0.9999	>0.9999	>0.9999	>0.9999	>0.9999

Supplementary Material Table S5. Gender-stratified comparison of leukocyte subsets and immune checkpoint markers in PBMCs and serum across minimal change disease (MCD), membranous nephropathy (MN), and healthy volunteers (HV).

	MCD Female	MCD Male	MN Female	MN Male	HV Female	HV Male	ALL	MCD Female vs. MCD Male	MCD Female vs. MN Female	MCD Female vs. MN Male	MCD Female vs. HV Female	MCD Female vs. HV Male	MCD Male vs. MN Female	MCD Male vs. MN Male	MCD Male vs. HV Female	MCD Male vs. HV Male	MN Female vs. MN Male	MN Female vs. HV Female	MN Female vs. HV Male	MN Male vs. HV Female	MN Male vs. HV Male	HV Female vs. HV Male
CD45+	97.22 (97.00-99.02)	98.34 (97.26-99.02)	96.94 (87.53-98.39)	98.29 (97.48-98.48)	97.36 (97.18-98.82)	99.03 (97.07-99.46)	0.2455	>0.9999	>0.9999	>0.9999	>0.9999	>0.9999	>0.9999	>0.9999	>0.9999	>0.9999	>0.9999	>0.9999	0.2255	>0.9999	>0.9999	>0.9999
CD3+	72.05 (70.18-80.37)	67.52 (64.86-71.35)	72.45 (68.58-75.06)	76.64 (64.58-78.87)	70.86 (67.52-71.75)	75.21 (72.98-79.03)	0.0351	0.4536	>0.9999	>0.9999	>0.9999	>0.9999	>0.9999	0.9344	>0.9999	0.0177	>0.9999	>0.9999	>0.9999	>0.9999	>0.9999	0.4877
CD19+	10.78 (8.67-12.81)	11.03 (6.37-19.08)	10.72 (9.56-11.88)	11.34 (9.17-13.43)	12.81 (8.94-13.95)	11.90 (6.97-14.84)	0.9743	>0.9999	>0.9999	>0.9999	>0.9999	>0.9999	>0.9999	>0.9999	>0.9999	>0.9999	>0.9999	>0.9999	>0.9999	>0.9999	>0.9999	>0.9999
CD3-CD16+CD56+	8.11 (6.03-15.38)	13.63 (8.28-20.76)	7.79 (5.39-12.73)	8.60 (7.67-15.19)	9.34 (9.19-23.54)	10.14 (7.59-13.74)	0.2527	>0.9999	>0.9999	>0.9999	>0.9999	>0.9999	0.6461	>0.9999	>0.9999	>0.9999	>0.9999	>0.9999	>0.9999	>0.9999	>0.9999	>0.9999
CD4+	40.39 (34.66-47.24)	41.07 (31.58-48.50)	41.08 (34.45-45.46)	45.92 (35.56-50.93)	39.08 (26.13-44.87)	47.08 (37.09-49.78)	0.4922	>0.9999	>0.9999	>0.9999	>0.9999	>0.9999	>0.9999	>0.9999	>0.9999	>0.9999	>0.9999	>0.9999	>0.9999	>0.9999	>0.9999	>0.9999
CD8+	27.11 (23.95-29.47)	22.35 (20.12-29.47)	29.20 (26.09-31.50)	24.69 (18.89-29.27)	27.11 (24.74-29.98)	26.32 (20.76-34.42)	0.2792	>0.9999	>0.9999	>0.9999	>0.9999	>0.9999	0.6902	>0.9999	>0.9999	>0.9999	>0.9999	>0.9999	>0.9999	>0.9999	>0.9999	>0.9999
Ratio CD4/CD8	1.51 (0.97-1.87)	2.04 (1.26-2.28)	1.36 (1.19-1.59)	1.69 (1.35-2.41)	1.44 (0.87-1.80)	1.80 (1.08-2.27)	0.5098	>0.9999	>0.9999	>0.9999	>0.9999	>0.9999	>0.9999	>0.9999	>0.9999	>0.9999	>0.9999	>0.9999	>0.9999	>0.9999	>0.9999	>0.9999

CD4+CTLA-4+	CD3-CD16+CD56+PD-L1+	CD19+PD-L1+	CD8+PD-L1+	CD4+PD-L1+	CD3-CD16+CD56+PD-1+	CD19+PD-1+	CD8+PD-1+	CD4+PD-1+
4.60 (2.00-5.91)	4.30 (3.71-4.77)	3.08 (2.55-4.75)	3.91 (2.35 -8.21)	6.45 (5.45-7.91)	6.12 (5.44-7.90)	2.22 (1.46-3.77)	17.33 (8.31-20.22)	9.19 (6.64-18.26)
3.02 (2.07-3.23)	5.16 (3.98-5.61)	4.69 (3.00-5.83)	5.75 (4.62-8.60)	6.06 (4.45-8.93)	5.73 (4.91-7.74)	2.28 (1.65-3.42)	19.45 (15.39-24.28)	12.25 (8.71-15.76)
6.28 (4.30-9.08)	2.34 (1.32-3.68)	2.95 (2.27-3.60)	4.50 (2.86-5.51)	13.46 (11.65-20.06)	6.73 (4.35-8.63)	2.35 (1.96-3.03)	11.44 (9.52-13.04)	8.77 (7.87-9.75)
4.90 (2.25 6.78)	3.19 (1.63-5.07)	4.52 (3.24-5.41)	3.27 (1.36-4.96)	15.73 (10.56-23.25)	5.47 (3.68-6.58)	2.70 (1.28-3.38)	9.54 (8.47-11.09)	8.96 (8.10-9.76)
7.33 (6.53-7.83)	3.19 (2.59-4.13)	0.49 (0.33-0.71)	0.37 (0.28-0.49)	1.25 (0.69-1.53)	3.02 (2.58-3.17)	0.24 (0.11-0.41)	1.47 (0.88-2.03)	0.77 (0.53-1.65)
6.97 (6.61-7.56)	3.77 (2.75-4.20)	0.78 (0.42-1.07)	0.54 (0.34-0.73)	1.05 (0.85-1.49)	2.84 (2.46-3.30)	0.27 (0.12-0.68)	1.65 (1.03-2.60)	0.99 (0.66-2.27)
<0.0001	0.0008	<0.0001	<0.0001	<0.0001	<0.0001	<0.0001	<0.0001	<0.0001
>0.9999	>0.9999	>0.9999	>0.9999	>0.9999	>0.9999	>0.9999	>0.9999	>0.9999
0.299	0.0476	>0.9999	>0.9999	0.1015	>0.9999	>0.9999	>0.9999	>0.9999
>0.9999	0.6726	>0.9999	>0.9999	0.06	>0.9999	>0.9999	>0.9999	>0.9999
0.0081	0.6346	0.0003	<0.0001	0.0132	0.0002	0.0001	<0.0001	<0.0001
0.0166	0.735	0.0008	<0.0001	0.0127	<0.0001	0.0002	<0.0001	<0.0001
0.0045	0.0024	>0.9999	>0.9999	0.0865	>0.9999	>0.9999	>0.9999	>0.9999
0.3604	0.0527	>0.9999	0.4765	0.0497	>0.9999	>0.9999	0.4234	>0.9999
<0.0001	0.0611	<0.0001	<0.0001	0.016	0.0027	0.0002	<0.0001	<0.0001
<0.0001	0.0618	<0.0001	<0.0001	0.0156	0.0005	0.0003	<0.0001	<0.0001
>0.9999	>0.9999	>0.9999	>0.9999	>0.9999	>0.9999	>0.9999	>0.9999	>0.9999
>0.9999	>0.9999	0.0028	0.0003	<0.0001	0.0044	0.0005	0.0013	0.001
>0.9999	>0.9999	0.0081	0.0018	<0.0001	0.0011	0.0006	0.0023	0.0017
0.0755	>0.9999	<0.0001	0.0016	<0.0001	0.0163	0.0004	0.001	<0.0001
0.1572	>0.9999	<0.0001	0.0087	<0.0001	0.0041	0.0005	0.0018	<0.0001
>0.9999	>0.9999	>0.9999	>0.9999	>0.9999	>0.9999	>0.9999	>0.9999	>0.9999

CD8+CD200+	CD4+CD200+	CD3-CD16+CD56+CD86+	CD19+CD86+	CD8+CD86+	CD4+CD86+	CD3-CD16+CD56+CTLA-4+	CD19+CTLA-4+	CD8+CTLA-4+
39.46 (36.74-41.23)	44.22 (39.00-47.62)	3.02 (2.04-3.95)	2.35 (1.41-2.41)	1.06 (0.50-2.45)	1.26 (0.78-1.82)	1.50 (1.00-2.17)	5.91 (3.90-7.03)	6.92 (5.88-8.10)
37.17 (33.91-41.50)	47.39 (41.05-50.48)	3.01 (1.81-4.22)	2.03 (1.66-2.75)	1.82 (1.26-2.58)	1.66 (1.16-2.11)	1.41 (0.88-2.81)	5.34 (4.03-6.13)	4.45 (4.02-5.68)
30.60 (25.89-35.12)	46.95 (44.32-52.16)	1.17 (1.00-1.84)	1.82 (1.57-2.62)	2.09 (1.77-2.46)	2.31 (1.82-2.75)	2.23 (1.06-2.76)	7.94 (6.97-8.70)	1.86 (1.38-4.00)
27.09 (19.77-31.70)	40.66 (36.05-51.50)	0.96 (0.74-2.06)	1.82 (1.52-2.57)	2.30 (1.86-2.71)	1.97 (1.54-2.55)	1.81 (0.89-2.41)	8.44 (6.77-9.62)	2.87 (2.10-3.75)
5.19 (4.53-5.51)	4.64 (4.27-4.99)	0.36 (0.21-1.46)	4.71 (3.74-5.38)	2.98 (2.02-3.31)	3.84 (3.68-4.25)	0.74 (0.30-1.62)	7.57 (6.62-8.14)	5.65 (5.45-5.86)
5.08 (4.46-5.41)	4.76 (4.67-5.12)	0.81 (0.59-1.28)	3.44 (2.46-3.97)	3.23 (2.30-4.51)	2.26 (1.07-3.56)	0.70 (0.30-1.12)	7.25 (6.36-7.86)	6.35 (5.63-6.58)
<0.0001	<0.0001	<0.0001	<0.0001	0.0026	<0.0001	0.0065	0.0005	<0.0001
>0.9999	>0.9999	>0.9999	>0.9999	>0.9999	>0.9999	>0.9999	>0.9999	0.0812
0.3482	>0.9999	0.1185	>0.9999	>0.9999	0.5735	>0.9999	0.0623	<0.0001
0.028	>0.9999	0.0134	>0.9999	0.498	>0.9999	>0.9999	0.0147	<0.0001
<0.0001	0.0002	<0.0001	<0.0001	0.0361	<0.0001	>0.9999	>0.9999	>0.9999
<0.0001	0.0003	0.0003	0.0027	0.0034	0.3482	0.2664	0.9769	>0.9999
0.797	>0.9999	0.0621	>0.9999	>0.9999	>0.9999	>0.9999	0.0184	0.3235
0.0926	>0.9999	0.0057	>0.9999	>0.9999	>0.9999	>0.9999	0.0032	0.4215
<0.0001	<0.0001	<0.0001	0.0003	0.5067	0.0002	0.8279	0.6694	>0.9999
<0.0001	<0.0001	<0.0001	0.0397	0.1009	>0.9999	0.0718	0.3723	0.2617
>0.9999	>0.9999	>0.9999	>0.9999	>0.9999	>0.9999	>0.9999	>0.9999	>0.9999
0.0115	<0.0001	0.947	0.0002	>0.9999	0.1105	0.3115	>0.9999	0.0054
0.003	<0.0001	>0.9999	0.0218	0.8148	>0.9999	0.0222	>0.9999	<0.0001
0.0264	<0.0001	>0.9999	<0.0001	>0.9999	0.0054	>0.9999	>0.9999	0.005
0.0063	0.0001	>0.9999	0.0016	>0.9999	>0.9999	0.1058	>0.9999	<0.0001
>0.9999	>0.9999	>0.9999	>0.9999	>0.9999	0.0546	>0.9999	>0.9999	>0.9999

sCTLA-4	sPD-L1	sPD-1	CD3-CD16+CD56+CD200R+	CD19+CD200R+	CD8+CD200R+	CD4+CD200R+	CD3-CD16+CD56+CD200+	CD19+CD200+
11.55 (8.14-12.06)	18.62 (13.12-19.46)	15.04 (13.27-18.27)	11.24 (7.42-12.59)	36.67 (35.59-40.29)	47.15 (45.30-54.48)	51.73 (44.33-55.94)	7.18 (5.54-8.81)	39.48 (30.83-46.17)
11.66 (9.81-12.23)	18.80 (15.82-19.72)	17.07 (14.52-18.86)	8.67 (6.87-11.50)	35.67 (34.25-39.69)	51.40 (48.93-55.15)	47.22 (45.86-54.20)	7.16 (5.98-8.33)	34.24 (32.15-39.76)
7.47 (6.22-7.85)	12.05 (10.03-12.65)	10.39 (9.33-12.40)	6.91 (6.35-8.15)	45.61 (42.98-49.22)	49.40 (45.10-53.11)	63.69 (59.03-68.68)	5.84 (5.56-7.68)	40.37 (37.81-51.29)
7.77 (6.48-8.33)	12.54 (10.45-13.44)	11.06 (9.36-12.13)	7.39 (5.82-8.30)	47.46 (42.63-53.54)	47.88 (42.42-52.48)	58.40 (55.17-77.67)	6.74 (4.50-8.35)	49.90 (39.57-54.32)
4.68 (4.41-4.98)	1.79 (1.68-1.85)	2.60 (2.44-2.68)	6.41 (5.44-7.61)	22.04 (11.22-34.43)	7.68 (6.11-9.45)	7.18 (6.51-8.08)	5.88 (4.78-6.23)	4.87 (4.54-5.23)
4.68 (4.41-5.10)	1.74 (1.66-1.85)	2.52 (2.40-2.68)	6.39 (5.44-7.09)	30.73 (23.76-35.19)	7.68 (5.76-8.91)	7.80 (5.39-8.66)	5.57 (4.87-6.35)	5.12 (4.81-5.48)
<0.0001	<0.0001	<0.0001	0.0003	<0.0001	<0.0001	<0,0001	0.03	<0.0001
>0.9999	>0.9999	>0.9999	>0.9999	>0.9999	>0.9999	>0.9999	>0.9999	>0.9999
0.1607	0.2975	0.1701	0.6451	0.0206	>0.9999	0.5668	>0.9999	>0.9999
0.2274	0.359	0.1025	0.1414	0.007	>0.9999	0.6727	>0.9999	>0.9999
<0.0001	<0.0001	<0.0001	0.0103	0.2526	0.0003	0.0028	0.7039	0.0005
<0.0001	<0.0001	<0.0001	0.0008	0.5112	<0.0001	0.0023	0.2073	0.0006
0.0311	0.0642	0.0513	>0.9999	0.0112	>0.9999	0.3097	>0.9999	>0.9999
0.0389	0.0672	0.0251	>0.9999	0.0034	>0.9999	0.3491	>0.9999	0.4073
<0.0001	<0.0001	<0.0001	0.1614	0.4013	<0.0001	0.0071	0.252	0.0025
<0.0001	<0.0001	<0.0001	0.0266	0.8006	<0.0001	0.0063	0.057	0.0035
>0.9999	>0.9999	>0.9999	>0.9999	>0.9999	>0.9999	>0,9999	>0.9999	>0.9999
0.1419	0.0655	0.0671	>0.9999	<0.0001	0.0003	<0.0001	>0.9999	<0.0001
0.0991	0.0179	0.0185	>0.9999	<0.0001	<0.0001	<0.0001	>0.9999	<0.0001
0.0181	0.0086	0.019	>0.9999	<0.0001	0.0004	<0.0001	>0.9999	<0.0001
0.0087	0.0012	0.0032	>0.9999	<0.0001	<0.001	<0.0001	>0.9999	<0.0001
>0.9999	>0.9999	>0.9999	>0.9999	>0.9999	>0.9999	>0.9999	>0.9999	>0.9999

qCD200R	qCD200	qCD86	qCTLA-4	qPD-L1	qPD-1	sCD200R	sCD200	sCD86
15.48 (9.93-18.85)	14.94 (12.97-20.05)	2.84 (2.09-4.71)	3.76 (3.00-5.06)	12.28 (9.64-14.84)	47.84 (19.53-57.51)	20.65 (19.29-22.76)	20.52 (19.37-21.60)	4.64 (4.32-4.78)
19.19 (11.93-22.98)	12.77 (5.11-18.19)	2.41 (1.50-3.28)	3.97 (1.89-4.46)	11.91 (9.27-14.97)	42.31 (26.44-57.35)	19.70 (17.76-20.74)	19.05 (17.72-20.85)	5.26 (4.42-5.63)
19.24 (16.42-24.24)	12.99 (7.99-15.94)	6.61 (4.11-7.98)	5.51 (3.96-7.04)	18.60 (13.67-26.47)	28.37 (22.39-42.01)	13.32 (12.69-14.04)	13.99 (13.22-14.83)	5.81 (5.32-6.36)
15.39 (10.86-20.88)	11.00 (9.25-15.28)	9.28 (6.82-10.83)	5.53 (4.80-6.46)	20.15 (15.89-27.59)	29.17 (18.15-39.46)	13.40 (11.63-15.42)	12.95 (12.12-13.74)	6.06 (5.31-6.61)
0.61 (0.28-3.16)	1.21 (0.32-2.10)	0.82 (0.41-1.92)	0.76 (0.26-2.51)	0.75 (0.51-1.83)	0.74 (0.41-1.07)	3.60 (3.40-3.83)	4.07 (3.84-4.33)	5.76 (5.43-6.41)
1.48 (0.56-2.23)	0.80 (0.59-2.53)	0.79 (0.40-3.03)	0.93 (0.62-1.99)	0.82 (0.55-1.47)	0.88 (0.37-3.61)	3.60 (3.40-3.93)	4.07 (3.84-4.44)	6.05 (4.99-6.49)
<0.0001	<0.0001	<0.0001	<0.0001	<0.0001	<0.0001	<0.0001	<0.0001	0.0005
>0.9999	>0.9999	>0.9999	>0.9999	>0.9999	>0.9999	>0.9999	>0.9999	>0.9999
>0.9999	>0.9999	>0.9999	>0.9999	>0.9999	>0.9999	0.0315	0.0642	0.0551
>0.9999	>0.9999	0.0717	>0.9999	0.274	>0.9999	0.0089	0.002	0.007
0.0007	<0.0001	0.1644	0.1048	0.0071	<0.0001	<0.0001	<0.0001	0.0263
0.0003	<0.0001	0.822	0.035	0.0024	<0.0001	<0.0001	<0.0001	0.0125
>0.9999	>0.9999	0.3534	0.8484	>0.9999	>0.9999	0.1193	0.2923	0.5264
>0.9999	>0.9999	0.0019	0.1665	0.1948	>0.9999	0.045	0.0176	0.1329
<0.0001	0.0002	>0.9999	0.6874	0.0109	<0.0001	<0.0001	<0.0001	0.3102
<0.0001	0.0004	>0.9999	0.3263	0.0039	<0.0001	<0.0001	<0.0001	0.2034
>0.9999	>0.9999	>0.9999	>0.9999	>0.9999	>0.9999	>0.9999	>0.9999	>0.9999
<0.0001	0.0004	0.0031	0.0028	<0.0001	0.0007	0.0482	0.0178	>0.9999
<0.0001	0.001	0.0214	0.0006	<0.0001	0.0002	0.0303	0.0101	>0.9999
0.0002	0.0002	<0.0001	<0.0001	<0.0001	0.0003	0.0196	0.0502	>0.9999
<0.0001	0.0005	<0.0001	<0.0001	<0.0001	<0.0001	0.0096	0.0279	>0.9999
>0.9999	>0.9999	>0.9999	>0.9999	>0.9999	>0.9999	>0.9999	>0.9999	>0.9999

Supplementary Material Table S6. Spearman's rank correlations for patients with minimal change disease (MCD).

Para zmiennych	R	t(N-2)	p
CD8+ & Ratio CD4/CD8	-0.858	-8.832	0.000
CD3+ & CD3-CD16+CD56+	-0.618	-4.157	0.000
HGB & CD19+CTLA-4+	-0.541	-3.404	0.002
CD4+ & CD8+	-0.513	-3.162	0.004
CD45+ & CD19+PD-L1+	-0.512	-3.157	0.004
IgA & Albumin	-0.494	-3.004	0.006
CD19+ & CD3-CD16+CD56+	-0.493	-2.999	0.006
Age & CD3-CD16+CD56+	-0.481	-2.905	0.007
Urea & CD8+PD-1+	-0.467	-2.791	0.009
LDL & CD19+	-0.460	-2.743	0.010
IgM & sPD-1	-0.457	-2.719	0.011
LYM & CD8+	-0.457	-2.717	0.011
MON & CD8+	-0.455	-2.704	0.012
Age & RBC	-0.454	-2.700	0.012
eGFR & qCTLA-4	-0.451	-2.676	0.012
Age & HGB	-0.449	-2.659	0.013
CD3-CD16+CD56+ & CD4+	-0.448	-2.649	0.013
EOS & CD4+CD200+	-0.444	-2.622	0.014
Age & eGFR	-0.436	-2.564	0.016
Total protein & CD8+PD-1+	-0.430	-2.523	0.018
CD4+CD200+ & CD8+CD200+	-0.430	-2.519	0.018
RBC & CD19+CTLA-4+	-0.429	-2.516	0.018
CD19+CD86+ & CD3-CD16+CD56+CD86+	-0.429	-2.513	0.018
Creatine & CD3-CD16+CD56+	-0.428	-2.508	0.018
PLT & qCD200R	-0.418	-2.432	0.022
Urea & CD8+PD-L1+	-0.413	-2.397	0.023
CD8+PD-1+ & CD3-CD16+CD56+PD-L1+	-0.411	-2.385	0.024
Cholesterol & CD19+PD-L1+	-0.410	-2.382	0.024
IgM & sCD86	-0.410	-2.378	0.024
Albumin & qCD200	-0.410	-2.376	0.025
LDL & CD19+PD-L1+	-0.408	-2.368	0.025

HGB & Total protein	-0.408	-2.363	0.025
HDL & CD3-CD16+CD56+PD-L1+	-0.407	-2.357	0.026
eGFR & sPD-L1	-0.403	-2.332	0.027
eGFR & sCTLA-4	-0.403	-2.332	0.027
Total protein & sCD86	-0.402	-2.324	0.028
CD19+ & CD8+	-0.401	-2.314	0.028
HGB & sCD200	-0.400	-2.309	0.029
Creatine & CD8+PD-L1+	-0.398	-2.295	0.029
IgA & CD8+CTLA-4+	-0.396	-2.284	0.030
Creatine & CD8+PD-1+	-0.386	-2.211	0.035
MON & CD19+CD86+	-0.383	-2.192	0.037
eGFR & CD19+PD-L1+	-0.382	-2.185	0.037
Uric acid & qCD200R	-0.377	-2.156	0.040
Trójglicerydy & CD8+CD200R+	-0.372	-2.119	0.043
CD8+ & CD8+CD86+	-0.371	-2.114	0.044
Age & HDL	-0.369	-2.103	0.045
IgM & qPD-1	-0.368	-2.097	0.045
LYM & EOS	-0.367	-2.089	0.046
CD45+ & CD8+CTLA-4+	-0.367	-2.085	0.046
CD4+PD-1+ & CD8+CTLA-4+	-0.366	-2.084	0.046
IgA & CD19+PD-1+	-0.366	-2.079	0.047
HGB & IgG	-0.364	-2.069	0.048
BAS & CD3-CD16+CD56+CD200+	-0.362	-2.056	0.049
HDL & CD3-CD16+CD56+CD200+	-0.361	-2.050	0.050
eGFR & CD8+	0.361	2.050	0.050
CD8+CD200R+ & sCD200	0.362	2.052	0.050
sPD-L1 & sCD200R	0.362	2.055	0.049
sCTLA-4 & sCD200R	0.362	2.055	0.049
CD8+PD-1+ & CD4+CD86+	0.364	2.069	0.048
WBC & sPD-L1	0.365	2.071	0.048
WBC & sCTLA-4	0.365	2.071	0.048
Urea & qPD-1	0.367	2.087	0.046
CD4+CTLA-4+ & CD8+CTLA-4+	0.367	2.090	0.046

WBC & IgA	0.370	2.107	0.044
LYM & CD4+	0.371	2.117	0.043
Uric acid & CD3+	0.372	2.120	0.043
MON & CD4+CD200+	0.374	2.133	0.042
Cholesterol & qCTLA-4	0.374	2.137	0.042
CD8+PD-1+ & CD4+PD-L1+	0.375	2.140	0.041
Total protein & CD8+	0.377	2.156	0.040
Urea & Ratio CD4/CD8	0.378	2.160	0.039
BAS & Cholesterol	0.379	2.167	0.039
CD3-CD16+CD56+ & CD8+PD-L1+	0.380	2.175	0.038
CD45+ & CD8+	0.381	2.182	0.038
Uric acid & CD4+CTLA-4+	0.383	2.196	0.037
HDL & qCD200	0.388	2.227	0.034
CD3+ & CD8+	0.389	2.233	0.034
RBC & CD8+CD86+	0.390	2.243	0.033
Urea & Uric acid	0.393	2.262	0.032
CD4+PD-L1+ & CD8+PD-L1+	0.393	2.263	0.032
Cholesterol & CD3-CD16+CD56+CTLA-4+	0.397	2.292	0.030
Total protein & CD3-CD16+CD56+PD-L1+	0.399	2.305	0.029
BAS & PLT	0.405	2.343	0.026
Proteinuria & CD3-CD16+CD56+CD200R+	0.405	2.345	0.026
CD8+PD-1+ & CD8+PD-L1+	0.407	2.360	0.025
CD3-CD16+CD56+ & CD8+PD-1+	0.408	2.368	0.025
Ratio CD4/CD8 & CD3-CD16+CD56+PD-L1+	0.409	2.372	0.025
BAS & HGB	0.411	2.386	0.024
LYM & CD4+CD200+	0.412	2.393	0.024
Urea & CD4+	0.415	2.417	0.022
NEU & RBC	0.416	2.418	0.022
PLT & CD4+CD200R+	0.416	2.423	0.022
PLT & Uric acid	0.423	2.468	0.020
MON & PLT	0.426	2.491	0.019
LDL & Proteinuria	0.426	2.494	0.019
Uric acid & HDL	0.429	2.515	0.018

Creatine & qCD200	0.430	2.517	0.018
HGB & CD8+CD86+	0.431	2.530	0.017
Albumin & CD4+CD200R+	0.432	2.533	0.017
CD3-CD16+CD56+CD200+ & sPD-1	0.434	2.550	0.017
Proteinuria & CD3-CD16+CD56+CTLA-4+	0.435	2.553	0.016
IgM & Total protein	0.437	2.571	0.016
WBC & CD3-CD16+CD56+PD-L1+	0.442	2.610	0.014
CD8+CTLA-4+ & CD19+CTLA-4+	0.446	2.634	0.014
PLT & Albumin	0.446	2.638	0.013
Age & Ratio CD4/CD8	0.447	2.643	0.013
Total protein & CD3+	0.450	2.664	0.013
CD4+CTLA-4+ & CD19+CTLA-4+	0.450	2.667	0.013
Albumin & CD3-CD16+CD56+PD-L1+	0.451	2.674	0.012
HGB & Cholesterol	0.451	2.677	0.012
sCD200 & qCD86	0.453	2.689	0.012
MON & Ratio CD4/CD8	0.459	2.734	0.011
RBC & LDL	0.465	2.783	0.010
LYM & Ratio CD4/CD8	0.471	2.825	0.009
BAS & LDL	0.474	2.851	0.008
HDL & CD8+PD-1+	0.482	2.911	0.007
CD19+PD-1+ & CD8+CTLA-4+	0.486	2.942	0.006
BAS & RBC	0.493	2.996	0.006
CD19+CD200+ & CD8+CD200R+	0.494	3.007	0.006
Age & CD4+	0.499	3.045	0.005
Age & Creatine	0.503	3.079	0.005
WBC & LYM	0.506	3.104	0.004
Total protein & Albumin	0.506	3.108	0.004
sPD-1 & sCD86	0.508	3.120	0.004
EOS & BAS	0.526	3.275	0.003
CD3+ & CD4+	0.527	3.280	0.003
EOS & CD8+CD200R+	0.527	3.281	0.003
qCD86 & qCD200	0.536	3.363	0.002
IgG & Albumin	0.546	3.452	0.002

Creatine & Uric acid	0.556	3.542	0.001
EOS & CD19+CD200+	0.557	3.548	0.001
LDL & CD3-CD16+CD56+CTLA-4+	0.558	3.561	0.001
NEU & MON	0.562	3.591	0.001
Urea & Proteinuria	0.562	3.591	0.001
IgG & IgM	0.562	3.592	0.001
eGFR & CD45+	0.584	3.804	0.001
Age & qPD-1	0.591	3.876	0.001
WBC & Trójglicerydy	0.598	3.952	0.000
Trójglicerydy & IgA	0.603	4.004	0.000
WBC & MON	0.604	4.011	0.000
Creatine & Proteinuria	0.616	4.136	0.000
NEU & LYM	0.623	4.210	0.000
RBC & HGB	0.630	4.298	0.000
Age & Urea	0.640	4.404	0.000
IgG & Total protein	0.724	5.551	0.000
Cholesterol & LDL	0.756	6.116	0.000
Urea & Creatine	0.827	7.781	0.000
CD4+ & Ratio CD4/CD8	0.850	8.547	0.000
MON & LYM	0.854	8.684	0.000

Supplementary Materials Table S7. Spearman's rank correlations for patients with membranous nephropathy (MN).

Para zmiennych	R	t(N-2)	p
CD8+ & Ratio CD4/CD8	-0.800	-7.063	0.000
EOS & IgM	-0.636	-4.365	0.000
CD3-CD16+CD56+PD-L1+ & CD3-CD16+CD56+CD200+	-0.634	-4.342	0.000
Urea & LDL	-0.562	-3.593	0.001
CD3-CD16+CD56+ & CD4+	-0.561	-3.583	0.001
Creatine & LDL	-0.560	-3.573	0.001
RBC & CD4+CD200R+	-0.551	-3.496	0.002
CD4+CD200R+ & sCD200R	-0.543	-3.422	0.002
Urea & Cholesterol	-0.537	-3.367	0.002

Creatine & CD8+CD200+	-0.531	-3.319	0.003
CD3-CD16+CD56+PD-L1+ & CD3-CD16+CD56+CD200R+	-0.509	-3.127	0.004
CD4+CD86+ & sCD86	-0.508	-3.120	0.004
IgM & IgA	-0.501	-3.066	0.005
CD4+CD200R+ & CD3-CD16+CD56+CD200R+	-0.499	-3.043	0.005
Creatine & eGFR	-0.487	-2.952	0.006
Proteinuria & CD4+CD200+	-0.478	-2.880	0.008
Creatine & Cholesterol	-0.473	-2.844	0.008
CD3-CD16+CD56+CD86+ & CD19+CD200+	-0.468	-2.799	0.009
CD8+ & CD19+CD200R+	-0.467	-2.797	0.009
IgA & qCD200	-0.464	-2.771	0.010
IgG & IgM	-0.460	-2.739	0.011
CD3-CD16+CD56+ & CD3-CD16+CD56+CD200R+	-0.459	-2.733	0.011
Proteinuria & CD4+CD200R+	-0.454	-2.697	0.012
Albumin & Proteinuria	-0.453	-2.691	0.012
CD8+ & CD4+PD-1+	-0.446	-2.639	0.013
IgA & qCD200R	-0.443	-2.618	0.014
CD3-CD16+CD56+ & Ratio CD4/CD8	-0.443	-2.615	0.014
Creatine & CD3-CD16+CD56+CD200R+	-0.442	-2.606	0.014
EOS & PLT	-0.439	-2.586	0.015
IgM & CD3-CD16+CD56+CTLA-4+	-0.439	-2.584	0.015
CD3+ & CD3-CD16+CD56+	-0.438	-2.581	0.015
PLT & Creatine	-0.437	-2.574	0.016
CD4+CD200R+ & sPD-L1	-0.433	-2.540	0.017
CD4+CD200R+ & sCTLA-4	-0.433	-2.540	0.017
IgG & CD8+CD86+	-0.431	-2.528	0.017
HGB & CD4+CD200+	-0.430	-2.521	0.018
Urea & CD8+CD200+	-0.428	-2.506	0.018
WBC & CD3-CD16+CD56+CTLA-4+	-0.428	-2.505	0.018
CD3+ & CD19+CD200R+	-0.428	-2.503	0.018
sCD86 & qCD200R	-0.426	-2.491	0.019
EOS & CD3-CD16+CD56+CD200+	-0.418	-2.433	0.022
CD8+ & qCD86	-0.418	-2.433	0.022

Age & LDL	-0.418	-2.432	0.022
CD19+ & CD8+	-0.417	-2.424	0.022
LDL & qCD86	-0.415	-2.413	0.023
HDL & qCD200	-0.409	-2.371	0.025
CD19+ & qPD-1	-0.408	-2.363	0.025
BAS & CD19+	-0.405	-2.345	0.026
CD19+CTLA-4+ & qCD200R	-0.405	-2.345	0.026
Age & Cholesterol	-0.405	-2.344	0.026
Urea & eGFR	-0.404	-2.339	0.027
RBC & CD4+CD200+	-0.403	-2.330	0.027
Age & sCD200	-0.401	-2.319	0.028
CD8+PD-L1+ & CD3-CD16+CD56+PD-L1+	-0.401	-2.317	0.028
CD8+PD-1+ & qCTLA-4	-0.397	-2.290	0.030
Cholesterol & IgG	-0.394	-2.271	0.031
Uric acid & CD3-CD16+CD56+CD200R+	-0.394	-2.265	0.031
IgG & qCD200	-0.392	-2.253	0.032
RBC & CD3-CD16+CD56+CTLA-4+	-0.387	-2.218	0.035
CD19+PD-L1+ & CD3-CD16+CD56+CD200R+	-0.383	-2.193	0.037
CD3-CD16+CD56+CD86+ & CD4+CD200+	-0.381	-2.182	0.038
Age & IgM	-0.376	-2.149	0.040
WBC & IgA	-0.376	-2.145	0.041
Trójglicerydy & Albumin	-0.372	-2.119	0.043
CD4+CTLA-4+ & qPD-1	-0.370	-2.107	0.044
Total protein & sCD200	-0.366	-2.080	0.047
CD45+ & CD19+CD200R+	-0.365	-2.077	0.047
CD3-CD16+CD56+CTLA-4+ & CD8+CD86+	-0.365	-2.076	0.047
Uric acid & Ratio CD4/CD8	-0.365	-2.072	0.048
Albumin & qPD-L1	-0.364	-2.071	0.048
MON & CD4+CTLA-4+	-0.364	-2.065	0.048
CD4+ & CD8+	-0.363	-2.062	0.049
Age & IgA	0.361	2.051	0.050
CD8+CD86+ & qPD-L1	0.361	2.051	0.050
Cholesterol & Ratio CD4/CD8	0.362	2.058	0.049

CD19+ & sCD200R	0.363	2.058	0.049
CD4+CD200+ & CD8+CD200+	0.363	2.063	0.048
IgA & CD19+CTLA-4+	0.364	2.069	0.048
WBC & CD3-CD16+CD56+CD86+	0.366	2.080	0.047
Trójglicerydy & CD3-CD16+CD56+	0.366	2.081	0.047
Proteinuria & CD4+	0.367	2.084	0.046
WBC & HGB	0.368	2.092	0.046
WBC & sPD-L1	0.368	2.092	0.046
WBC & sCTLA-4	0.368	2.092	0.046
Trójglicerydy & Proteinuria	0.368	2.093	0.046
MON & IgG	0.369	2.102	0.045
HGB & qPD-L1	0.370	2.107	0.044
Trójglicerydy & CD4+PD-L1+	0.371	2.113	0.044
Trójglicerydy & CD19+PD-L1+	0.374	2.137	0.042
Albumin & CD4+CD200R+	0.375	2.141	0.041
CD19+CD200+ & CD8+CD200R+	0.377	2.154	0.040
RBC & CD4+	0.377	2.157	0.040
IgM & qCD200	0.378	2.161	0.039
Urea & IgG	0.378	2.161	0.039
EOS & IgA	0.378	2.164	0.039
HDL & Albumin	0.379	2.167	0.039
IgG & Total protein	0.379	2.167	0.039
sCD200 & qCD200R	0.382	2.184	0.037
Cholesterol & CD8+CD86+	0.382	2.186	0.037
HGB & Uric acid	0.383	2.192	0.037
Ratio CD4/CD8 & qPD-L1	0.384	2.201	0.036
PLT & HDL	0.386	2.211	0.035
NEU & CD3-CD16+CD56+	0.389	2.231	0.034
Urea & Total protein	0.390	2.240	0.033
Cholesterol & Proteinuria	0.392	2.253	0.032
Cholesterol & Trójglicerydy	0.392	2.256	0.032
CD3-CD16+CD56+CD200R+ & sCD200R	0.393	2.259	0.032
Cholesterol & qPD-L1	0.394	2.265	0.031

Trójglicerydy & CD8+CD86+	0.394	2.269	0.031
BAS & CD3-CD16+CD56+CD86+	0.396	2.279	0.030
CD4+ & qPD-L1	0.398	2.295	0.029
BAS & Uric acid	0.399	2.302	0.029
sPD-L1 & sCD200R	0.402	2.320	0.028
sCTLA-4 & sCD200R	0.402	2.320	0.028
qPD-L1 & qCTLA-4	0.402	2.320	0.028
MON & CD3-CD16+CD56+PD-L1+	0.402	2.322	0.028
NEU & MON	0.402	2.324	0.028
WBC & BAS	0.405	2.346	0.026
PLT & LDL	0.409	2.373	0.025
CD19+CD200R+ & qPD-L1	0.412	2.391	0.024
EOS & IgG	0.412	2.395	0.024
Urea & Uric acid	0.413	2.396	0.023
RBC & eGFR	0.414	2.403	0.023
CD19+CTLA-4+ & CD3-CD16+CD56+CTLA-4+	0.416	2.423	0.022
sCD200 & qPD-L1	0.418	2.438	0.021
MON & BAS	0.419	2.445	0.021
BAS & CD3-CD16+CD56+	0.420	2.446	0.021
CD8+PD-L1+ & CD3-CD16+CD56+CD200+	0.421	2.459	0.020
Cholesterol & CD3+	0.422	2.462	0.020
WBC & CD3-CD16+CD56+	0.427	2.497	0.019
CD3-CD16+CD56+CD86+ & sCD200	0.428	2.507	0.018
Cholesterol & sCD200	0.430	2.520	0.018
CD19+ & CD19+CD200R+	0.432	2.536	0.017
MON & qPD-1	0.436	2.563	0.016
IgM & CD8+CD86+	0.437	2.571	0.016
RBC & qPD-L1	0.439	2.582	0.015
Cholesterol & CD3-CD16+CD56+CD200R+	0.442	2.604	0.015
Ratio CD4/CD8 & CD4+PD-1+	0.444	2.620	0.014
Ratio CD4/CD8 & qCD86	0.454	2.695	0.012
LYM & CD3-CD16+CD56+CD86+	0.460	2.741	0.011
CD19+ & qPD-L1	0.461	2.747	0.010

eGFR & LDL	0.472	2.831	0.008
Uric acid & CD3-CD16+CD56+	0.474	2.848	0.008
sPD-1 & sPD-L1	0.477	2.870	0.008
sPD-1 & sCTLA-4	0.477	2.870	0.008
Total protein & CD4+CD200R+	0.482	2.910	0.007
CD19+PD-L1+ & CD4+CD200R+	0.483	2.915	0.007
HGB & CD45+	0.484	2.925	0.007
CD19+PD-1+ & CD4+PD-L1+	0.484	2.929	0.007
CD45+ & CD3+	0.489	2.963	0.006
CD4+PD-1+ & sPD-L1	0.491	2.981	0.006
CD19+ & qCTLA-4	0.499	3.050	0.005
HGB & Proteinuria	0.500	3.055	0.005
RBC & Cholesterol	0.508	3.121	0.004
Creatine & Uric acid	0.514	3.168	0.004
Proteinuria & CD45+	0.519	3.216	0.003
eGFR & Cholesterol	0.520	3.218	0.003
Creatine & CD3-CD16+CD56+	0.520	3.219	0.003
Cholesterol & CD4+	0.521	3.226	0.003
NEU & Uric acid	0.522	3.242	0.003
BAS & Urea	0.532	3.322	0.002
RBC & CD45+	0.534	3.344	0.002
LDL & CD3-CD16+CD56+CD200R+	0.544	3.434	0.002
EOS & Urea	0.550	3.481	0.002
BAS & Creatine	0.563	3.606	0.001
BAS & CD45+	0.564	3.615	0.001
RBC & Trójglicerydy	0.566	3.636	0.001
HGB & Trójglicerydy	0.567	3.641	0.001
MON & CD3-CD16+CD56+	0.604	4.014	0.000
RBC & Proteinuria	0.609	4.066	0.000
Proteinuria & RBC	0.609	4.066	0.000
LYM & sPD-L1	0.648	4.498	0.000
LYM & sCTLA-4	0.648	4.498	0.000
PLT & eGFR	0.666	4.725	0.000

NEU & LYM	0.670	4.778	0.000
CD3+ & CD4+	0.677	4.869	0.000
WBC & NEU	0.679	4.893	0.000
WBC & LYM	0.681	4.918	0.000
Urea & Creatine	0.772	6.425	0.000
CD4+ & Ratio CD4/CD8	0.806	7.214	0.000
Total protein & Albumin	0.848	8.478	0.000
Cholesterol & LDL	0.852	8.622	0.000
RBC & HGB	0.902	11.059	0.000

Supplementary Materials Table S8. ROC

	CD4+PD-1+		
	MCD vs. MN	MCD vs. HV	MN vs. HV
Area	0.6289	1	1
Std. Error	0.08278	0	0
95% confidence interval	0.4666 to 0.7911	1.000 to 1.000	1.000 to 1.000
P value	0.0863	<0.0001	<0.0001
	CD8+PD-1+		
	MCD vs. MN	MCD vs. HV	MN vs. HV
Area	0.755	0.9867	1
Std. Error	0.07547	0.01005	0
95% confidence interval	0.6071 to 0.9029	0.9670 to 1.000	1.000 to 1.000
P value	0.0007	<0.0001	<0.0001
	CD19+PD-1+		
	MCD vs. MN	MCD vs. HV	MN vs. HV
Area	0.5233	0.96	0.9428

Std. Error	0.07558	0.02111	0.02805
95% confidence interval	0.3752 to 0.6715	0.9186 to 1.000	0.8878 to 0.9977
P value	0.7562	<0.0001	<0.0001
	CD3-CD16+CD56+PD-1+		
	MCD vs. MN	MCD vs. HV	MN vs. HV
Area	0.5644	0.9578	0.8767
Std. Error	0.07496	0.02259	0.05008
95% confidence interval	0.4175 to 0.7114	0.9135 to 1.000	0.7785 to 0.9748
P value	0.3912	<0.0001	<0.0001
	CD4+PD-L1+		
	MCD vs. MN	MCD vs. HV	MN vs. HV
Area	0.95	1	1
Std. Error	0.02416	0	0
95% confidence interval	0.9026 to 0.9974	1.000 to 1.000	1.000 to 1.000
P value	<0.0001	<0.0001	<0.0001
	CD8+PD-L1+		
	MCD vs. MN	MCD vs. HV	MN vs. HV
Area	0.7017	0.9794	0.9639
Std. Error	0.06858	0.01794	0.02034
95% confidence interval	0.5673 to 0.8361	0.9443 to 1.000	0.9240 to 1.000
P value	0.0073	<0.0001	<0.0001
	CD19+PD-L1+		
	MCD vs. MN	MCD vs. HV	MN vs. HV
Area	0.5378	0.9733	0.99
Std. Error	0.0752	0.02184	0.008589
95% confidence interval	0.3904 to 0.6852	0.9305 to 1.000	0.9732 to 1.000
P value	0.6152	<0.0001	<0.0001
	CD3-CD16+CD56+PD-L1+		
	MCD vs. MN	MCD vs. HV	MN vs. HV

Area	0.7611	0.7978	0.5833
Std. Error	0.06466	0.05597	0.07857
95% confidence interval	0.6344 to 0.8878	0.6881 to 0.9075	0.4293 to 0.7373
P value	0.0005	<0.0001	0.2675
	CD4+CTLA-4+		
	MCD vs. MN	MCD vs. HV	MN vs. HV
Area	0.6994	0.9556	0.6717
Std. Error	0.06897	0.0255	0.08184
95% confidence interval	0.5643 to 0.8346	0.9056 to 1.000	0.5113 to 0.8321
P value	0.008	<0.0001	0.0224
	CD8+CTLA-4+		
	MCD vs. MN	MCD vs. HV	MN vs. HV
Area	0.8478	0.5222	0.98
Std. Error	0.05578	0.08047	0.01323
95% confidence interval	0.7385 to 0.9571	0.3645 to 0.6799	0.9541 to 1.000
P value	<0.0001	0.7675	<0.0001
	CD19+CTLA-4+		
	MCD vs. MN	MCD vs. HV	MN vs. HV
Area	0.8172	0.7389	0.6772
Std. Error	0.05509	0.06707	0.07243
95% confidence interval	0.7093 to 0.9252	0.6074 to 0.8703	0.5353 to 0.8192
P value	<0.0001	0.0015	0.0184
	CD3-CD16+CD56+CTLA-4+		
	MCD vs. MN	MCD vs. HV	MN vs. HV
Area	0.5283	0.73	0.7661
Std. Error	0.0766	0.06464	0.06281
95% confidence interval	0.3782 to 0.6785	0.6033 to 0.8567	0.6430 to 0.8892
P value	0.7062	0.0022	0.0004
	CD4+CD86+		

	MCD vs. MN	MCD vs. HV	MN vs. HV
Area	0.6994	0.7989	0.7122
Std. Error	0.0676	0.06031	0.07354
95% confidence interval	0.5669 to 0.8319	0.6807 to 0.9171	0.5681 to 0.8564
P value	0.008	<0.0001	0.0047
	CD8+CD86+		
	MCD vs. MN	MCD vs. HV	MN vs. HV
Area	0.6772	0.7789	0.6928
Std. Error	0.07141	0.06063	0.07438
95% confidence interval	0.5373 to 0.8172	0.6601 to 0.8977	0.5470 to 0.8386
P value	0.0184	0.0002	0.0103
	CD19+CD86+		
	MCD vs. MN	MCD vs. HV	MN vs. HV
Area	0.5289	0.9244	0.9283
Std. Error	0.07688	0.03235	0.03129
95% confidence interval	0.3782 to 0.6796	0.8610 to 0.9879	0.8670 to 0.9897
P value	0.7007	<0.0001	<0.0001
	CD3-CD16+CD56+CD86+		
	MCD vs. MN	MCD vs. HV	MN vs. HV
Area	0.8611	0.9578	0.6794
Std. Error	0.04556	0.02243	0.07019
95% confidence interval	0.7718 to 0.9504	0.9138 to 1.000	0.5419 to 0.8170
P value	<0.0001	<0.0001	0.017
	CD4+CD200R+		
	MCD vs. MN	MCD vs. HV	MN vs. HV
Area	0.8433	1	1
Std. Error	0.05293	0	0
95% confidence interval	0.7396 to 0.9471	1.000 to 1.000	1.000 to 1.000
P value	<0.0001	<0.0001	<0.0001

	CD8+CD200R+		
	MCD vs. MN	MCD vs. HV	MN vs. HV
Area	0.5728	1	1
Std. Error	0.07473	0	0
95% confidence interval	0.4263 to 0.7192	1.000 to 1.000	1.000 to 1.000
P value	0.3329	<0.0001	<0.0001
	CD19+CD200R+		
	MCD vs. MN	MCD vs. HV	MN vs. HV
Area	0.9611	0.8222	1
Std. Error	0.02035	0.05433	0
95% confidence interval	0.9212 to 1.000	0.7157 to 0.9287	1.000 to 1.000
P value	<0.0001	<0.0001	<0.0001
	CD3-CD16+CD56+CD200R+		
	MCD vs. MN	MCD vs. HV	MN vs. HV
Area	0.7311	0.8217	0.6761
Std. Error	0.06683	0.0543	0.06885
95% confidence interval	0.6001 to 0.8621	0.7152 to 0.9281	0.5412 to 0.8110
P value	0.0021	<0.0001	0.0191
	CD4+CD200+		
	MCD vs. MN	MCD vs. HV	MN vs. HV
Area	0.5111	1	1
Std. Error	0.07711	0	0
95% confidence interval	0.3600 to 0.6622	1.000 to 1.000	1.000 to 1.000
P value	0.8825	<0.0001	<0.0001
	CD8+CD200+		
	MCD vs. MN	MCD vs. HV	MN vs. HV
Area	0.9089	1	1
Std. Error	0.03555	0	0
95% confidence interval	0.8392 to 0.9786	1.000 to 1.000	1.000 to 1.000

P value	<0.0001	<0.0001	<0.0001
	CD19+CD200+		
	MCD vs. MN	MCD vs. HV	MN vs. HV
Area	0.7517	1	1
Std. Error	0.06306	0	0
95% confidence interval	0.6281 to 0.8753	1.000 to 1.000	1.000 to 1.000
P value	0.0008	<0.0001	<0.0001
	CD3-CD16+CD56+CD200+		
	MCD vs. MN	MCD vs. HV	MN vs. HV
Area	0.6167	0.78	0.6044
Std. Error	0.07291	0.06137	0.07861
95% confidence interval	0.4738 to 0.7596	0.6597 to 0.9003	0.4504 to 0.7585
P value	0.1206	0.0002	0.1646
	sPD-1		
	MCD vs. MN	MCD vs. HV	MN vs. HV
Area	0.9511	1	1
Std. Error	0.02389	0	0
95% confidence interval	0.9043 to 0.9979	1.000 to 1.000	1.000 to 1.000
P value	<0.0001	<0.0001	<0.0001
	sPD-L1		
	MCD vs. MN	MCD vs. HV	MN vs. HV
Area	0,9078	1	1
Std. Error	0.03801	0	0
95% confidence interval	0.8333 to 0.9823	1.000 to 1.000	1.000 to 1.000
P value	<0.0001	<0.0001	<0.0001
	sCTLA-4		
	MCD vs. MN	MCD vs. HV	MN vs. HV
Area	0.9078	1	0.9389
Std. Error	0.03801	0	0.02814

95% confidence interval	0.8333 to 0.9823	1.000 to 1.000	0.8837 to 0.9941
P value	<0.0001	<0.0001	<0.0001
	sCD86		
	MCD vs. MN	MCD vs. HV	MN vs. HV
Area	0.7989	0.7989	0.5
Std. Error	0.05747	0.05747	0.07515
95% confidence interval	0.6862 to 0.9115	0.6862 to 0.9115	0.3527 to 0.6473
P value	<0.0001	<0.0001	>0.9999
	sCD200		
	MCD vs. MN	MCD vs. HV	MN vs. HV
Area	0.9978	1	1
Std. Error	0.003051	0	0
95% confidence interval	0.9918 to 1.000	1.000 to 1.000	1.000 to 1.000
P value	<0.0001	<0.0001	<0.0001
	sCD200R		
	MCD vs. MN	MCD vs. HV	MN vs. HV
Area	0.9856	1	1
Std. Error	0.01081	0	0
95% confidence interval	0.9644 to 1.000	1.000 to 1.000	1.000 to 1.000
P value	<0.0001	<0.0001	<0.0001
	qPD-1		
	MCD vs. MN	MCD vs. HV	MN vs. HV
Area	0.5944	0.9911	0.9989
Std. Error	0.07649	0.00817	0.001882
95% confidence interval	0.4445 to 0.7444	0.9751 to 1.000	0.9952 to 1.000
P value	0.2089	<0.0001	<0.0001
	qPD-L1		
	MCD vs. MN	MCD vs. HV	MN vs. HV
Area	0.8356	0.9889	0.9989
Std. Error	0.05044	0.01164	0.001882

95% confidence interval	0.7367 to 0.9344	0.9661 to 1.000	0.9952 to 1.000
P value	<0.0001	<0.0001	<0.0001
	qCTLA-4		
	MCD vs. MN	MCD vs. HV	MN vs. HV
Area	0.7644	0.83	0.9067
Std. Error	0.06288	0.05368	0.04097
95% confidence interval	0.6412 to 0.8877	0.7248 to 0.9352	0.8264 to 0.9870
P value	0.0004	<0.0001	<0.0001
	qCD86		
	MCD vs. MN	MCD vs. HV	MN vs. HV
Area	0.8561	0.7539	0.8933
Std. Error	0.05708	0.06661	0.04494
95% confidence interval	0.7442 to 0.9680	0.6233 to 0.8844	0.8053 to 0.9814
P value	<0.0001	0.0007	<0.0001
	qCD200		
	MCD vs. MN	MCD vs. HV	MN vs. HV
Area	0.6011	0.9756	0.9756
Std. Error	0.075	0.0156	0.01706
95% confidence interval	0.4541 to 0.7481	0.9450 to 1.000	0.9421 to 1.000
P value	0.1785	<0.0001	<0.0001
	qCD200R		
	MCD vs. MN	MCD vs. HV	MN vs. HV
Area	0.5311	0.9878	0.97
Std. Error	0.07557	0.01008	0.01756
95% confidence interval	0.3830 to 0.6792	0.9680 to 1.000	0.9356 to 1.000
P value	0.6789	<0.0001	<0.0001

Supplementary Materials Table S9. Table of antibodies used, including catalog numbers and antibody suppliers.

**Antigen/Target**	**Clone**	**Fluorochrome**	**Catalog number**	Location	Location
CD3	HIT3a	BV510	564713	BD Biosciences	Franklin Lakes, NJ, USA
CD4	OKT4	APC-R700	566808	BD Biosciences	Franklin Lakes, NJ, USA
CD8	HIT8a	BV605	569169	BD Biosciences	Franklin Lakes, NJ, USA
CD16	3G8	BV650	563692	BD Biosciences	Franklin Lakes, NJ, USA
CD19	SJ25C1	PerCP	332780	BD Biosciences	Franklin Lakes, NJ, USA
CD56	NCAM16.2	BV650	564057	BD Biosciences	Franklin Lakes, NJ, USA
PD-1	MIH4	PE	557946	BD Biosciences	Franklin Lakes, NJ, USA
PD-L1	MIH1	APC	563741	BD Biosciences	Franklin Lakes, NJ, USA
CTLA-4	BNI3	PE	560939	BD Biosciences	Franklin Lakes, NJ, USA
CD86	BU63	APC	571636	BD Biosciences	Franklin Lakes, NJ, USA
CD200	MRC OX-104	PE	552475	BD Biosciences	Franklin Lakes, NJ, USA
CD200R	OX-108	APC	329308	BioLegend	San Diego, CA, USA

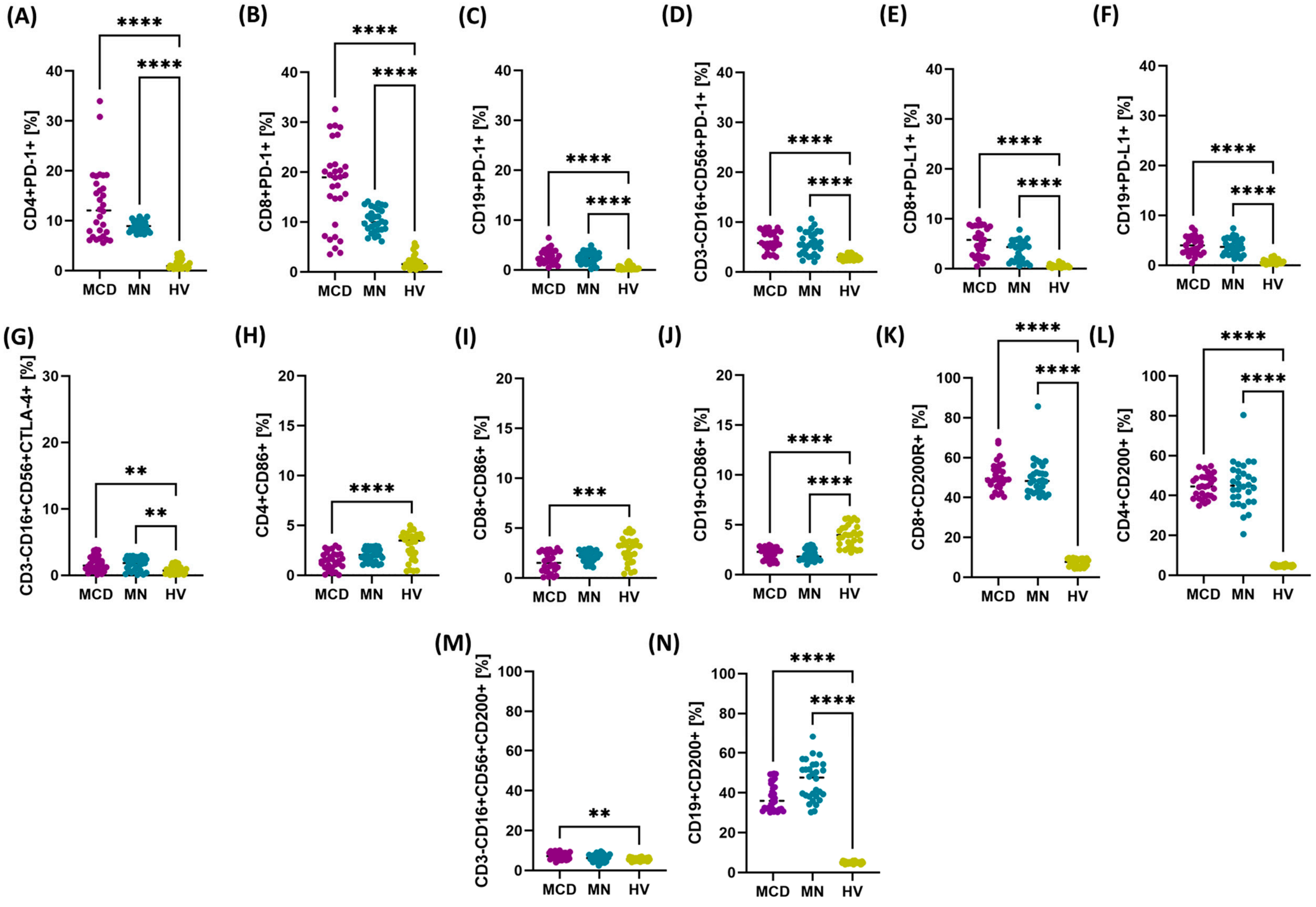

Supplementary Materials Figure S1. Percentage of PD-1, PD-L1, CTLA-4, CD86, CD200, and CD200R-positive cells in major lymphocyte and NK subpopulations in peripheral blood of newly diagnosed, untreated patients with minimal change disease (MCD) and membranous nephropathy (MN), and healthy volunteers (HV). Dot plots show individual values with medians indicated (horizontal line). Panels: (A) CD4+PD-1+, (B) CD8+PD-1+, (C) CD19+PD-1+, (D) CD3-CD16+CD56+PD-1+ (NK), (E) CD8+PD-L1+, (F) CD19+PD-L1+, (G) CD3-CD16+CD56+CTLA-4+ (NK), (H) CD4+CD86+, (I) CD8+CD86+, (J) CD19+CD86+, (K) CD8^{+}CD200R+, (L) CD4+CD200+, (M) CD3-CD16+CD56+CD200+ (NK), (N) CD19+CD200+. Notations: $p \geq 0.05$, * $p < 0.05$, ** $p < 0.01$, *** $p < 0.001$, **** $p < 0.0001$.

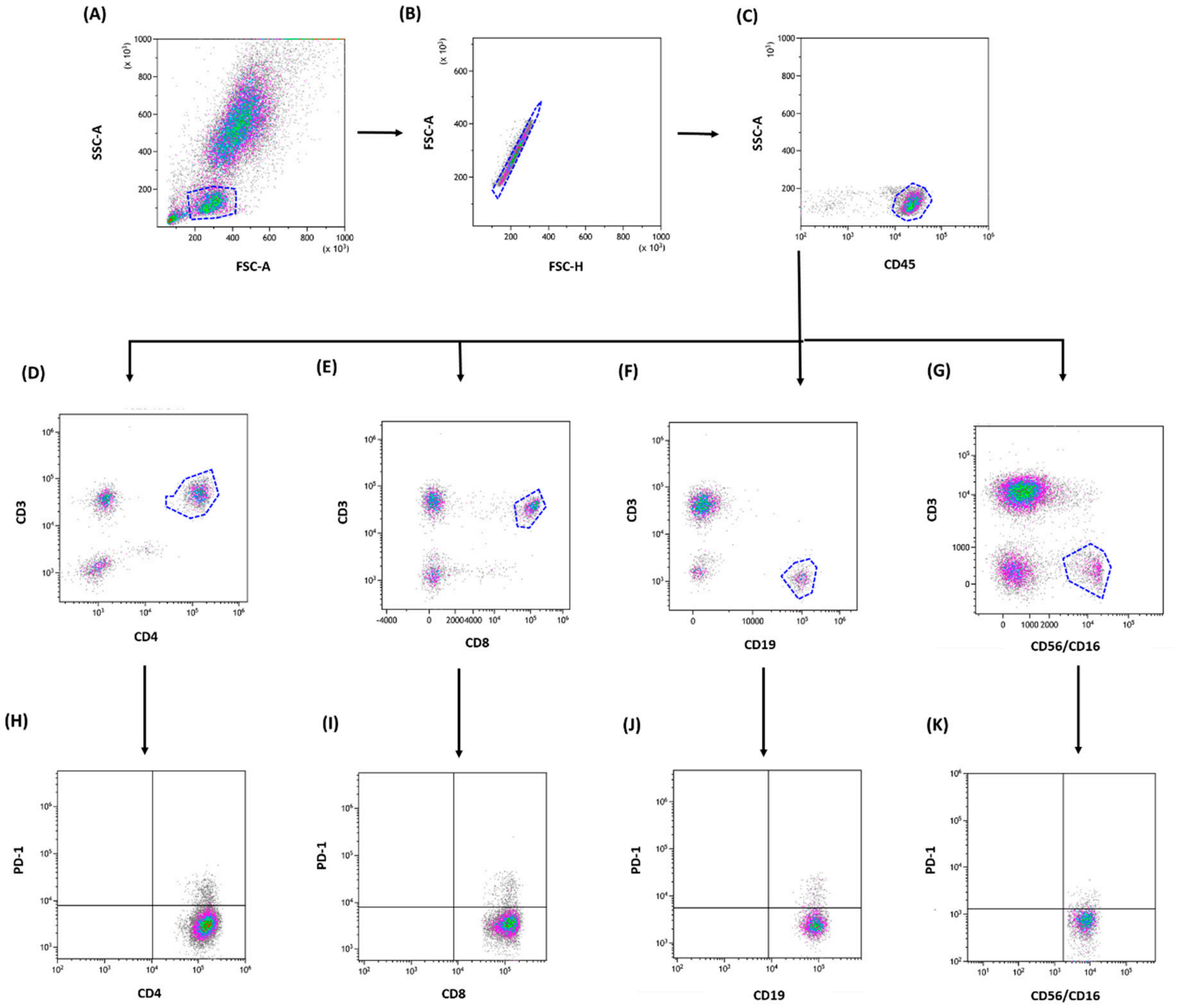

Supplementary Materials Figure S2 Schematic representation of the gating strategy used in the flow cytometric analysis of peripheral blood mononuclear cells (PBMC). In panel (A), the lymphocyte population was separated based on light scatter parameters (FSC-A/SSC-A), whereas in panel (B), a gate on singlets (FSC-H vs. FSC-A) was applied to exclude doublets. In panel (C), CD45^high leukocytes with low SSC-A were identified, allowing for the rejection of extracellular fragments and cellular debris. From this master gate, the subsequent lymphocyte populations shown in panels (D–G) were derived: (D) CD3^+CD4^+ helper T cells, (E) CD3^+CD8^+ cytotoxic T cells, (F) CD3^{-}CD19^+ B cells, and (G) NK cells defined as CD3^{-}CD56/CD16^+ lymphocytes. CD56 and CD16 markers were labeled with a cocktail of antibodies conjugated to the same fluorochrome, therefore the horizontal axis in panel (G) reflects the combined CD56/CD16 signal, and the NK gate includes only CD3^{-} events with a high signal in this channel. Panels (H–K) show the final analysis of PD-1 checkpoint receptor expression within individual subpopulations: (H) CD4^+PD-1^+ T cells, (I) CD8^+PD-1^+ T cells, (J) CD19^+PD-1^+ B cells, and (K) CD56/CD16^+PD-1^+ NK cells.
